# Effect of Nanofillers on Properties and Pervaporation Performance of Nanocomposite Membranes: A Review

**DOI:** 10.3390/membranes12121232

**Published:** 2022-12-05

**Authors:** Hamideh Sardarabadi, Shirin Kiani, Hamed Karkhanechi, Seyed Mahmoud Mousavi, Ehsan Saljoughi, Hideto Matsuyama

**Affiliations:** 1 Department of Chemical Engineering, Faculty of Engineering, Ferdowsi University of Mashhad, Mashhad 9177948974, Iran; 2 Research Center for Membrane and Film Technology, Department of Chemical Science and Engineering, Kobe University, 1-1 Rokkodai, Nada-ku, Kobe 657-8501, Japan

**Keywords:** review, nanofillers, pervaporation, nanocomposite membranes, nanotechnology

## Abstract

In recent years, a well-known membrane-based process called pervaporation (PV), has attracted remarkable attention due to its advantages for selective separation of a wide variety of liquid mixtures. However, some restrictions of polymeric membranes have led to research studies on developing membranes for efficient separation in the PV process. Recent studies have focused on preparation of nanocomposite membranes as an effective method to improve both selectivity and permeability of polymeric membranes. The present study provides a review of PV nanocomposite membranes for various applications. In this review, recent developments in the field of nanocomposite membranes, including the fabrication methods, characterization, and PV performance, are summarized.

## 1. Introduction

In recent years, many industries (including chemical, pharmaceutical, and food industries) have been forced to improve their current separation technologies into more efficient ones to minimize waste and recover valuable products from waste streams. [1,2]. In this regard, research activities dealing with membrane-based separation processes have experienced rapid growth among separation processes developed so far [2]. In particular, a well-known membrane-based process for selective separation of a wide variety of liquid mixtures called pervaporation (PV), has attracted remarkable attention in recent years [3,4,5]. PV offers many advantages over other present separation processes such as low operating cost, reduced energy demand, environmentally friendly operation, and membranes with adjustable separation properties suitable for a wide variety of applications [6,7].

**Figure 1:**
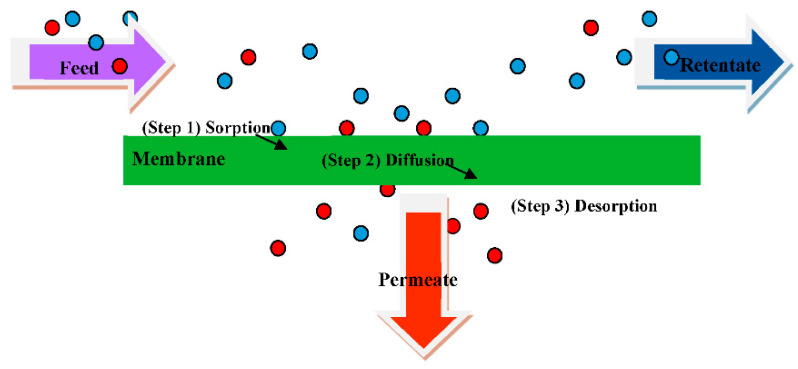
Schematic representation of solution-diffusion mechanism through a membrane.

To identify the ideal membrane material and design the preferred membrane structure, a better understanding of the mass transfer mechanism is essential [8]. In the PV process, the feed liquid mixture contacts one side of a membrane. Thereby, the minor component preferentially transports through the membrane. The mass transfer is accompanied by a phase change from liquid to vapor due to the lower vapor pressure on the permeate side than the feed side of the membrane [7,9,10,11]. In detail, the transport through PV membranes typically occurs according to a solution-diffusion mechanism [6,12]. Accordingly, permeation in the PV process is a result of preferential sorption of a component on the membrane surface, diffusion through the membrane, and desorption on the permeate side [6,8,13,14]. Figure 1 the solution-diffusion mechanism through the membrane in three steps in which separation occurs by PV. It is important to note that the separation performance of PV membranes can be determined by solubility, sorption, and diffusivity of permeants in the membrane in terms of permeation rate (flux) and selectivity (separation factor) [15].

In general, applications of the PV process may be classified into the following three categories [4,5,11,16,17,18,19]:

- Dehydration of aqueous-organic mixtures;
- Organics removal from aqueous-organic mixtures; and
- Separation of organic-organic mixtures.

It is important to realize that separation of liquid mixtures by PV is based on membrane affinity to the minor component in terms of its hydrophilic, hydrophobic, or organophilic nature [12,20]. Moreover, it should be noted that the membrane is the key element to achieve success in the PV process since its material determines separation performance [3,5,16,21,22,23]. Generally, an ideal PV membrane should possess a thin top layer skin (without any defects) and excellent sorption capacity for the desired solute, low swelling to maintain selectivity and structural stability, good mechanical strength, chemical, and thermal stability, high permeability without compromising selectivity, and economic viability [6,16]. However, the nonporous structure of the PV membrane leads to reductions in the flux values which limits the industrial application areas. It should be noted that the dehydration of organic liquids is the main industrial application of PV. Poly (vinyl alcohol) (PVA), polyimides, polymaleimides, poly (acrylonitrile) (PAN), chitosan (CS), and Nafion are some hydrophilic polymers used as membranes for pervaporation dehydration. Among these, PVA-based membranes are used on an industrial scale in the PV process [24,25].

As the need for more efficient membranes is critical to PV technology, developing a suitable membrane material with high performance (i.e., high permeation flux and high separation factor) is important [3,21]. Numerous studies have been carried out on PV membranes to prepare an optimized membrane material to maximize separation performance [26]. There are several modification methods to improve separation performance of membranes in the PV process. These include blending with another polymer, cross-linking, and incorporating nanoparticles in the polymer structure [7,21,26,27]. Among these modification methods, incorporation of nanoparticles in the organic polymer matrix is the most practical method to develop new membranes called nanocomposite membranes [21,26,28,29,30]. Nanocomposite membranes have immense potential to improve permeation performance in PV technology [29,30]. Recent studies have focused on PV nanocomposite membranes due to advantages such as high strength, high thermal and chemical stability, and enhancement in permeation barrier properties of polymers [26,27,31,32,33,34]. Improved properties are probably caused by combination of individual properties of the polymer and nano-sized particles, as well as the interaction between the materials in the nanoscale structure [35,36,37]. However, in spite of the above advantages of nanocomposite membranes, there are a few limitations which cannot be neglected. One of the main limitations of these membranes is the effective mixing of two dissimilar phases since the nanoparticles usually tend to aggregate in a polymer matrix to reduce high surface energy. The interfacial interaction between nanomaterials and the polymer is an important factor which could control the reinforcing efficiency of nanocomposite membrane. Functionalization of the nanomaterials using several chemical groups could improve their homogenization inside the polymer matrix [38].

The current research interest in the field of nanocomposite membranes and PV tends to idealize, overvalue, and overestimate the performance of nanocomposite membranes regarding their industrial scaling up [39]. There are some studies on the nanomaterials in nanocomposite membranes to overcome these limitations which are promising, however, more studies are needed on this issue [24].

**Figure 2:**
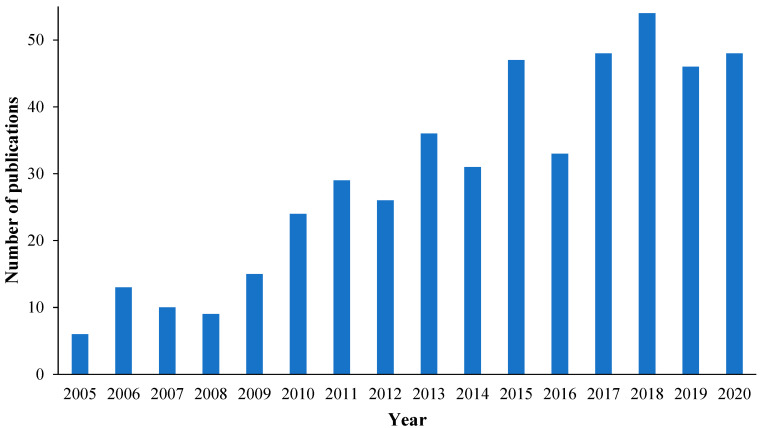
Annual Number of publications from 2005 to 2020 retrieved via the keyword “nanocomposite” and “pervaporation” in the topic of the paper, as reported by Web of Science (http://apps.webofknowledge.com, 15 June 2021).

The researchers’ interest in developing PV nanocomposite membranes is reflected by the number of publications regarding this subject between 2005–2020. As illustrated in Figure 2, the number of publications increased erratically while there was a marked improvement in 2018.

Although nanocomposite membranes have been the subject of numerous published papers, their application in the PV process has not been reviewed yet. Therefore, the present article aimed to provide a comprehensive review of recent research studies conducted on PV nanocomposite membranes. In detail, this review is focused on preparation methods of PV nanocomposite membranes and the effects of nanoparticles on membrane properties, as well as PV separation performance. Furthermore, the present review allows a better comparison of the results of numerous studies in the field of PV nanocomposite membranes which can be very useful for future studies to obtain reliable experimental data.

## 2. Nanocomposite Membranes Used in the PV Process

Recently, polymer nanocomposites have attracted increasing attention from both industry and academia due to the feasibility of improving their properties by combination of nanostructured materials and polymer matrix [26,34,36]. Specifically, organic-inorganic nanocomposite membranes have been the subject of intensive investigations due to their potential applications in membrane separation processes [36,40].

Polymeric membranes are widely used in the PV process on account of their low cost as well as their easy production with the possibility of scale-up [7,11,16,41,42]. However, many of these polymeric membranes are not suitable for harsh chemical and high-temperature environments because of their low chemical and thermal resistance [7,16,43]. Moreover, another drawback is their moderate permeability and separation capability [16]. On the other hand, inorganic materials as membranes offer several advantages over polymeric membranes such as superior chemical resistance, thermal stability, and good separation performance [7,16]. However, restrictions such as complicated and expensive manufacturing procedures along with their low mechanical stability limit the application of inorganic materials in membrane preparation [7,16]. Incorporation of nano-sized inorganic material into organic polymers has been proved as an attractive method of merging the best characteristics of two phases while potentially overcoming their limitations in the PV separation process [7,21,32,36,44]. Moreover, using this method, a trade-off between productivity and selectivity of polymeric membranes can be overcome [16].

**Figure 3:**
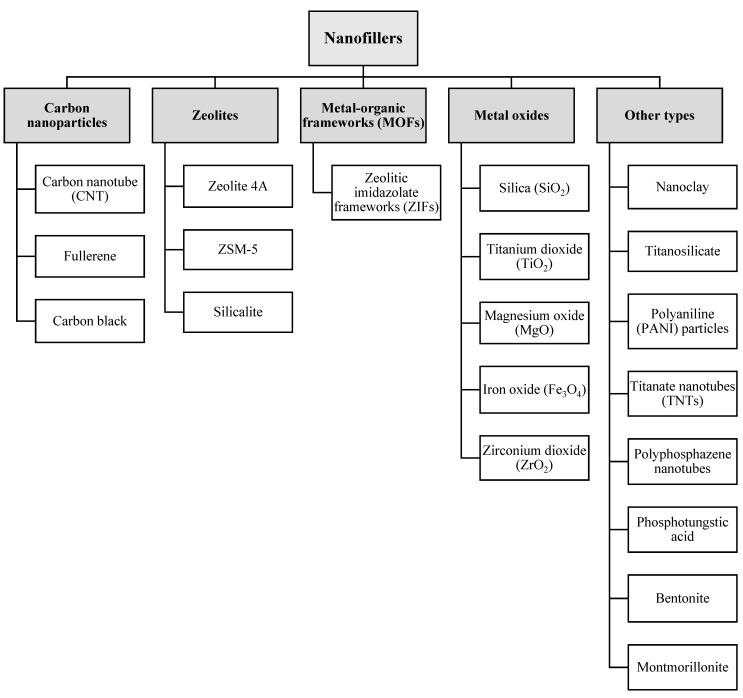
Different types of nanofillers applied in the preparation of nanocomposite PV membranes.

Over the past decades, a plethora of nanostructured materials including carbon nanoparticles [27,29,30,34,42,45,46,47,48,49,50,51,52,53,54,55,56], zeolites [5,7,10,12,57,58,59,60,61], metal-organic frameworks (MOFs) [62,63,64,65,66,67,68,69,70], metal oxides [3,4,7,22,26,28,36,41,45,71,72,73,74,75,76,77,78,79,80,81,82,83,84,85,86,87,88,89,90,91,92], titanosilicate [21], nano-clay [32,93,94,95,96], nano-sized polyaniline (PANI) particles [97,98], polyphosphazene nanotubes (PZSNTs) [99], titanate nanotubes (TNTs) [44], and phosphotungstic acid (PTA) [13,33] have been used as fillers in the polymer matrix to fabricate nanocomposite membranes with higher PV separation performance. Figure 3 gives a brief account of different types of nanofillers applied in the preparation of PV nanocomposite membranes.

PV nanocomposite membranes may be grouped into three main classes based on the permeate properties [8,15,16]: (1) Hydrophilic membranes; (2) Hydrophobic membranes; (3) Target-organophilic membranes. Table 1 summarizes some PV nanocomposite membranes which have been investigated with emphasis given to type of application.

## 3. Fabrication Methods of Nanocomposite Membranes

Two methods have been employed in preparing nanocomposite membranes: bottom-up or top-down. The first method is a chemical process in which precursors are utilized to grow from nano-sized to well-ordered structures, such as the sol-gel method, and top-down methods use physical techniques to disperse nanoparticles in the polymer matrix to get the desirable material [43].

The conventional preparation techniques to fabricate nanocomposites membranes that have been used for the PV process include (1) solution blending, (2) polymerization (in situ and interfacial), and (3) sol-gel process [43]. The preparation methods of PV nanocomposite membranes reported in the literature are presented in the following sections.

### 3.1. Solution Blending

Solution blending is the most common method to fabricate polymeric nanocomposite membranes [43,102]. This method can be used for all kinds of nanoparticles, and it is easy to control polymer and nanoparticles concentration [43,127]. However, the disadvantage of this method is the aggregation of nanoparticles in the membrane structure [43,127]. To improve membrane properties by this method, proper dispersion of nanomaterials in the polymer matrix is required [128].

The first step of the solution blending method is preparation of a homogeneous mixture of polymer and nanoparticles by thoroughly stirring and sonication to prevent the nanoparticles from agglomeration [8,43]. There are three techniques to prepare a mixed solution [43]:

(a) Dispersion of nanoparticles into the solvent and addition of polymer
(b) Polymer dissolution in the solvent and the addition of nanoparticles
(c) Dispersion of nanoparticles and mixing it with a solution of the polymer in the solvent

In the next step of solution casting the polymer solution is spread onto a flat surface to form a thin film followed by removal of the solvent by evaporation or phase inversion processes [8,43]. There are several ways to achieve phase inversion for membrane preparation such as wet casting, dry casting, or dry-wet casting processes. PV membranes should be either entirely dense or asymmetric with a thin dense selective layer [43]. In the wet casting process, the cast film is immersed in the non-solvent bath for solvent removal, and asymmetric membranes with interconnected cell structures are obtained [8,16,129]. The dry casting method, in which the solvent is removed from the film by the evaporation process, can be used to prepare dense membranes [8,16]. For dry-wet casting, a highly volatile solvent is used in the casting solution since it includes an evaporation step before phase inversion that promotes formation of a top dense layer [8,16,129].

**Figure 4:**
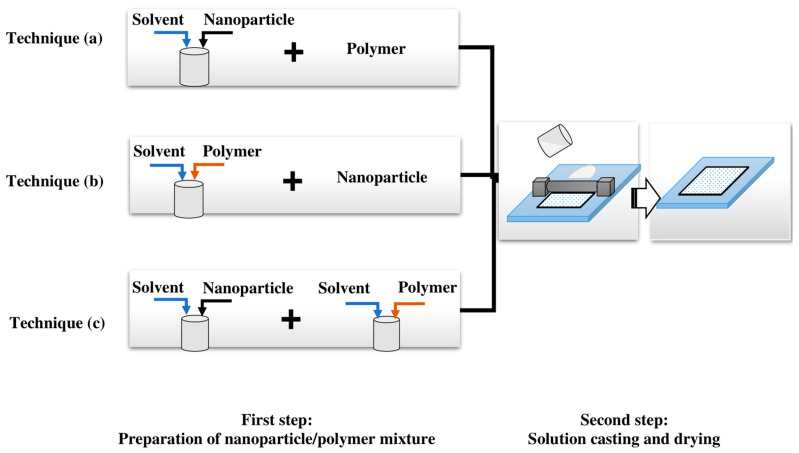
Schematic representation of steps involved in the preparation of nanocomposite PV membranes. Technique (**a**): Dispersion of nanoparticles into the solvent and addition of polymer; Technique (**b**): Polymer dissolution in the solvent and the addition of nanoparticles; Technique (**c**): Dispersion of nanoparticles and mixing it with a solution of the polymer in the solvent.

It should be noted that membrane morphology can change by variations in drying temperature and environment [129]. When a slow evaporation process is used for solvent removal, polymer chains which were stretched through casting can be detached from each other. Consequently, a loosely packed structure will be formed [3]. On the other hand, in fast solvent evaporation, immediate vitrification of polymer chains occurs in their stretched state, and a packed structure will be formed [3]. Moreover, it has been reported that a dense surface can be formed at a high evaporation rate of solvent and non-solvent [129]. Figure 4 shows a schematic diagram of the solution blending method.

Among the studies reported, the majority of researchers have employed solution blending to prepare PV nanocomposite membranes. Membranes prepared using this method are summarized in Table 2. It should be noted that some publications have not reported details of the membrane fabrication procedure.

In most of the studies, the cast film was allowed to dry at room temperature initially and then heated in a vacuum oven to evaporate the remaining solvent in the film to prepare the PV nanocomposite membranes [12,21].

One of the significant challenges for preparation of nanocomposite membranes by the solution casting technique is development of voids between polymer and nanoparticle phases [3]. Thus, some researchers have attempted to modify the membrane fabrication procedure to prepare membranes with higher separation efficiency [3]. For instance, Jiang et al. [3] fabricated the PI/MgO nanocomposite membrane for pervaporative dehydration of isopropanol (IPA) aqueous solution by employing high temperatures during membrane formation to eliminate the voids between the two phases. In their study, the membrane was formed at high temperatures under a vacuum after solution casting. It was found that a high-temperature casting environment (above T_g_) has advantages such as fast evaporation of solvent during membrane formation that can prevent sedimentation and aggregation of nanoparticles, and intimate contact between polymer chains and nanoparticles [3]. In another study, Bakhtiari et al. [7] prepared nanocomposite PI membranes using nano-sized aerosil SiO_2_ and nano-sized zeolite for pervaporative separation of the IPA/water mixtures. The films were peeled from the glass plate at a high temperature in order to avoid film rupturing during peeling. Their results showed no voids formed around incorporated nanoparticles and nearly uniform distribution without serious agglomeration of nanoparticles inside the polymer matrix was observed. Besides, proper links between polymers and nanoparticles were attributed to membrane preparation at higher temperatures [7]. The micro topographical changes in ZIF/PDMS membranes produced by the sonication-enhanced assembly were investigated by Yan et al. [69]. They found that compared to the membranes fabricated by the traditional method, the selective layer of membrane had a much more uniform thickness due to good dispersion of nanoparticles within the polymer matrix. In addition, the square roughness of the nanocomposite membranes decreased because sonication prohibited formation of aggregates of nanoparticles [69].

In an interesting work, Shi et al. [66] synthesized nano-sized ZIF and prepared PBI/ZIF nanocomposite membranes with uniform morphology by a modified dry casting method for PV dehydration of ethanol, IPA, and butanol. After solution casting, the obtained films were dried in a vacuum oven followed by cooling down in two steps. The membranes were solvent-exchanged with methanol to remove the residual solvent and dried once more in the vacuum oven [66].

Hu et al. [46] have conducted a study on the preparation of nanocomposite membranes including polyvinyl amine (PVAm), PVA, and MWCNTs using the solution coating technique for dehydration of ethylene glycol by PV. In this technique, a blend solution containing polymers and MWCNTs is placed into a glass bottle, and a microporous polysulfone substrate is placed inside the bottle cap. After that, they are assembled and the membrane coating solution was attached to the substrate. Then, the bottle assembly is heated in an oven at 55 °C [46].

Recently, Yan et al. [69] employed the sonication-enhanced in situ assembly method to prepare hybrid PDMS/SiO_2_ and PDMS/ZIF membranes with a high degree of dispersion of nanoparticles. At first, a suspension of PDMS and nanoparticles are ultrasonicated, and simultaneously a porous substrate is immersed in the suspension such that the suspension coats the substrate surface. In detail, the sonication could help prevent aggregation of nanoparticles on the supporting surface. Afterward, the substrate is rotated to remove the solvent from the surface of the film. A lamp was held on the other side of the film to accelerate this removal. The resultant films were dried at ambient temperature initially to remove the remaining solvent and then they were placed in a convection oven at 80 °C to crosslink the PDMS solution [69].

### 3.2. In Situ and Interfacial Polymerization

In the in situ polymerization method, nanoparticles are dispersed into organic monomers, and then the mixture is polymerized by adding a proper catalyst [43,131]. The nanoparticles can be linked with polymer chains by covalent bonds, which enable good dispersion of nanofillers inside the polymer matrix [131]. However, using this method cannot prevent the aggregation of nanoparticles in the resultant nanocomposite membranes [131]. Table 3 gives a summary of PV nanocomposite membranes prepared by the polymerization method.

Naidu et al. [97] prepared PVA/PANI hybrid nanocomposite membrane by in situ polymerization of aniline into the PVA matrix in acidic media. To prepare a nanocomposite structure, aniline monomer was introduced into the PVA matrix, and an aqueous solution of ammonium persulfate was added to this mixture to obtain the colloidal PANI particles. After casting the resultant solution, the dried membranes were cross-linked with glutaraldehyde [97].

A well-known technique for preparation of thin composite membranes is interfacial polymerization of reactive monomers on the surface of a microporous support film, which can be used for preparation of PV membranes [8,43]. This technique leads to formation of a thin selective layer on top of the substrate which can significantly enhance the membrane flux [8]. Moreover, the selection of suitable monomers for interfacial polymerization can improve chemical resistance, thermal stability, and long-term durability of the selective layer [8].

Fathizadeh et al. [10] synthesized nano NaX zeolite/PA thin film nanocomposite membrane over polyacrylonitrile (PAN) support by interfacial polymerization for use in PV dehydration of aqueous alcohol solutions. They first synthesized nano NaX zeolite by a hydrothermal reaction and then dispersion in toluene solution. Thin-film nanocomposite membrane was prepared by immersion of PAN support in an aqueous solution of m-phenylenediamine (MPD) and subsequently into toluene solution of trimesoylchloride (TMC) and nano NaX zeolite to form an active top layer on PAN support [10].

Wang et al. [37] utilized a direct polycondensation method to fabricate PA/clay hybrid nanocomposite membrane for separation of aqueous ethanol mixtures by PV. The hybrid polymers were prepared by direct polycondensation of 4,4′-methylenedianiline (MDA) and 4,4′-hexafluoroisopropylidenedibenzoic acid (6FDAc) in the presence of organo-clay (sodium dodecyl sulfate (SDS)-clay) in *N*-methyl-2-pyrrolidinone (NMP) solvent. Membrane characterization showed the SDS-clay aggregates in the PA structure, which implied that the silicate layers could not be exfoliated into the PA structure [37].

### 3.3. Sol-Gel Method

The sol-gel technique is widely used for preparation of polymer-inorganic nanocomposite membranes [43]. In this method, polymers, oligomers or organic monomers and inorganic precursors are mixed in the solution to obtain a morphology exhibiting co-continuity of phases. After that, the inorganic precursors hydrolyze and condense, so small particles can nucleate and precipitate out to form well-dispersed nano-metric particles in the polymer matrix [131,133]. Table 4 lists nanocomposite membranes prepared using the sol-gel fabrication method for PV application.

Compared to the two methods mentioned earlier, this method offers advantages, such as moderate reaction conditions, high chemical homogeneity, and controlled morphology. It should be noted that hybridization can lead to better dimensional stability of the membranes which is considered as another advantage of this method [43,131]. On the other hand, the disadvantage is that it causes more shrinkage and a lower number of voids [43].

### 3.4. Other Methods

Other fabrication methods have successfully been used to prepare PV nanocomposite membranes. In this section, a review of the different methods of preparation of PV nanocomposite membranes reported in the literature is provided Table 5.

In a study done by Penkova et al. [29], poly (phenylene isophthalamide)/CNTs nanocomposite membranes prepared by solid-phase interaction for pervaporative separation of methanol/methyl tert-butyl ether (MTBE) mixture. In this study, polymer and nanotube powders were mixed in a porcelain mortar, and the composites were dissolved in the solvent after the solid-phase interaction (N, *N*-dimethylacetamide (DMAc)). Unfortunately, the membrane characterizations did not reveal any strong interaction between CNTs and polymer. Moreover, presence of large agglomerates in the membrane indicated that CNTs have not been dispersed well [29].

In another work, Liu et al. [58] fabricated silicalite-PDMS nanocomposite membrane on capillary support using a packing-filling method for bio-butanol recovery by PV. To prepare these ultrathin homogeneous membranes, in the first step called “packing”, a porous alumina capillary support was coated by silicalite-1 nanocrystals using the dip-coating technique. At the second step called “filling”, the interspaces among the nanocrystals were filled with the PDMS phase. After that, the membrane was dried at room temperature and then drying was continued at 50 °C under vacuum [58].

In an interesting work, Ong et al. [48] prepared a green nanocomposite membrane composed of PHB-functionalized MWCNTs and CS for dehydration of 1,4-dioxane by PV. In their study, the MWCNTs were first functionalized with PHB and then aligned into a PTFE membrane filter template through a filtration process before being incorporated into the CS matrix. After bulk alignment, a solution casting technique was used to cast the CS onto the template to form nanocomposite membranes. It was reported that bulk alignment could optimize the reinforcing effect of functionalized MWCNTs (PHB-MWCNT) and reduce the required amount of MWCNTs to achieve the desired properties [48].

In a work conducted by Liu et al. [64], a novel plugging-filling method was successfully used to fabricate a homogeneous ZIF/PMPS silicone rubber nanocomposite membrane on a HOSSM for efficient recovery of furfural from dilute aqueous solution by PV. For the preparation of these nanocomposite membranes, at first, the holes in the top layer of the HOSSM were plugged with ZIF-8 nanoparticles. After that, by dip-coating, the ZIF-plugged HOSSM with PMPS, the spaces between the nanoparticles and mesh wires were filled with silicone rubber [64]. The homogeneous HOSSM/ZIF-8/PMPS nanocomposite membrane exhibited excellent performance and good stability because of space restriction and physical cross-linking of the HOSSM. Furthermore, fabricating membranes using this method allowed nanoparticles to create preferential pathways for furfural molecules causing in higher selectivity and permeability compared to that of the membranes fabricated by the solution blending method [64,134].

Li et al. [74] used a new approach called the layer-by-layer assembly method. To prepare nanohybrid multilayer membranes comprising ZrO_2_ and Al_2_O_3_ nanoparticles and PECs such as poly (diallyl dimethyl ammonium chloride) (PDDA)/PSS and poly (ethyleneimine)/polyacrylic acid for the pervaporative separation of acetone-water mixtures. Single-component nanoparticles were incorporated into both the polycation and polyanion layers using this method. Assembly of nanohybrid multilayer was successfully formed on both flat sheets and hollow fiber porous substrates. For preparation of hollow fiber nanohybrid multilayer membranes, a crossflow negative pressure filtration cell was used to carry out the assembly experiments. PDDA-coated ZrO_2_ and PSS-coated ZrO_2_ aqueous solution passed through hollow fibers using two peristaltic pumps and a vacuum pump to create a negative pressure outside the hollow fiber. After the assembly process, the resultant membranes were washed with water, and then they were dried by a nitrogen gas stream [74].

Zhang et al. [70] fabricated MOF/PVA nanohybrid membranes on a ceramic tubular substrate through a typical pressure-driven assembly method for PV separation of toluene/n-heptane mixtures. At first, the Cu_3_(BTC)_2_/PVA solution (BTC = benzene-1,3,5-tricarboxylate) was sonicated to obtain a stable homogeneous dispersion. At the same time, the tubular ceramic substrates were immersed in a silane coupling agent solution. Then, a rubber plug was placed on a vessel under vacuum, and the Cu_3_(BTC)_2_/PVA solution was poured into the vessel to assemble it onto amino ceramics substrate. Finally, the resultant membranes were dried in an oven at 40 °C [70].

## 4. Effect of the Nanoparticles on Membrane Properties

Up to now, the reported studies have demonstrated that the incorporation of nanomaterials into polymers could tune the membrane structure and other properties of membranes such as swelling degree and hydrophilicity/hydrophobicity. The main focus of this section is on the effects caused when nanofillers are added to the membrane matrix.

### 4.1. Membrane Structure

There are numerous factors which affect membrane separation performance including chemical structure and membrane morphology which play essential roles [83]. Based on available studies on nanocomposite membranes, introduction of nanoparticles into the polymer matrix induces changes in membrane structure, including free volume (the total fraction of void spaces in the membrane), and crystallinity.

#### 4.1.1. Membrane Free Volume

Various studies have been carried out to investigate the effects of nanoparticles on nanocomposite membrane structure. Based on reported research papers, addition of nanofillers into membranes affects the polymer chain packing, and hence free volume will change in the membrane [28,69,80]. Introduction of nanofillers into membrane has two possible effects on polymer structure as well as membrane morphology which subsequently affects permeability and selectivity of the nanocomposite membranes [6]. Generally, for glassy polymers, it has been reported that addition of nonporous fillers often increases membrane free volume due to disruption of polymer chain packing. On the contrary, in the rubbery polymers like PDMS, nonporous fillers could be easily accommodated within its flexible chains and the free volume of the polymer will not increase [135].

When the nanomaterials disrupt the normal polymer chain packing, the free volume of the membrane increases [26,28,83,104]. In this case, as the properties of polymer phase differs from the fillers and also due to the strong aggregation tendency of the fillers, some defects form at the polymer–nanofiller interface due to the weak adhesion between polymer and nanofillers [136]. Usually, if rigid or nonporous nanofillers such as CNTs were dispersed in a polymer, the available free volume and consequently molecular diffusion in the polymer matrix will increase (higher permeability). However, the size-separating nature of the polymer will be weakened (lower selectivity) [6]. For instance, CS-wrapped CNTs/PVA nanocomposite PV membranes were studied by Peng et al. [34]. Using positron annihilation lifetime spectroscopy (PALS) experiments, it was confirmed that the incorporation of CNTs into the membrane can tune the chain packing of the polymer so that the free volume increases.

It should be noted that PALS is a standard method to study the free volume characteristics of the nanocomposite membranes in which the nanosized voids within the polymer matrix can be quantitatively measured [137]. In a very recent study carried out by Ding et al. [126], the free volume parameters of modified graphitic carbon nitride nanosheets/PEBA membranes on the PSf support layer were characterized by the PALs method. It was found that the Cu^+^ and Fe^2+^ ions co-impregnated carbon nitride interrupted the packing of polymer chains so that the interface between the PEBA matrix and fillers increased. It was also reported that the interactions between PEBA and nanosheets restricted the mobility of polymer chains which in turn contributed to formation of membranes with compact structure without non-selective interfacial defects. At higher filler loading (7 wt.%), the agglomerated nanosheets caused a decrease in the interfacial region. Naidu et al. [97] calculated the fractional free volume of PANI nanoparticles/PVA nanocomposite membranes at different nanofiller loadings and found that with increasing nanoparticles, the free volume space increases as a result of morphological changes in the nanocomposite membranes. Liu et al. [44] evaluated the internal free volume properties of TNTs-embedded CS nanocomposite membranes applying the PALS technique. It was found that the free volume fraction of nanocomposite membranes increased compared to pure CS membrane. A similar conclusion was drawn in a study done by Razavi et al. [138] in which an increase in the fractional free volume of the nanocomposite membrane was reported. Hua et al. [63] investigated the effect of incorporation of ZIF nanoparticles on polymer chain packing as well as the fractional free volume of membranes. The results revealed that the fractional free volume increased with an increase in ZIF loading. Alberto et al. [125] fabricated organophilic membranes comprising of PIM and reduced amine-functionalized GO fillers for the recovery of organic solvents from aqueous solutions by PV. Scanning electron microscopy (SEM) images of the cross-section of membranes illustrated that the addition of graphene-like fillers affected morphology of membranes at the microscale. However, they reported that SEM analysis could not clarify the changes in the packing of polymer chains at the molecular level. This study reported that the incorporation of fillers caused an increase in free volume due to the creation of voids at the filler/polymer interface. Gao et al. [139] investigated the characteristics of polymer-MOF nanocomposite membranes for PV separation, and their results revealed that the voids at the interface between PEK-c and ZIF nanocrystals could act as channels for permeate diffusion through the membrane. Narkkun et al. [100] prepared double-network PVA nanocomposite membranes and reported that the free volume of polymer matrix increased because of the presence of silica nanospheres in the polymer matrix which disrupted the interactions among the polymer chains. In another study [62], the effects of using ZIF nanoparticles in the PVA matrix were investigated. It was found that the incorporation of nanoparticles into the PVA matrix at lower content led to an increase in membrane free volume by disrupting the inherent organization of polymer chains. Even so, it was revealed that by increasing ZIF loading in the nanocomposite membranes, membrane free volume decreased due to partial rigidification, while at high ZIF content, polymer compactness was reduced because of aggregation of ZIF particles.

It should be noted that the non-selective voids which are formed at the nanoparticle-polymer interface may induce lower selectivity and higher permeation flux of membranes [26,28]. To overcome this problem, surface modification of nanofillers is an effective way to enhance their compatibility and create more favorable transport properties, because surface properties of nanofillers can influence their interactions with polymer matrix [26]. For example, in a study done by Gao et al. [56], MWCNT decorated by hydrophilic nanoparticles (Fe_3_O_4_ nanoparticles) were incorporated into SA to modify hydrophilicity of nanofillers and improve their dispersion in polymer matrix. It was found that the decorated Fe_3_O_4_ increased free volume in the SA matrix and consequently more diffusion pathways were created for permeation of the penetrant molecules. Moreover, they reported that when polymer chains were adsorbed onto the surface of inorganic particles, their mobility decreased due to the interaction between polymer and inorganic particles. In another study, Peng et al. [34] prepared a novel nanocomposite membrane composed of CS-wrapped MWCNT and PVA polymer. CS was used to prevent aggregation of CNTs in PVA matrix. Free volume characteristics of the membranes were investigated, and it was found that incorporation of CS-wrapped CNTs could tune the packing of hydrophilic chains in the interface, increase PVA free volume, and provide internal nanochannels for permeation.

Nanomaterials may also decrease the polymer free volume. Polymer–fillers interfacial adhesion is so strong that the free volume reduces near the fillers surface. This is known as polymer rigidification as a consequence of uniform stresses that arise during membrane formation process [136]. Since the rigidified polymer layer around the fillers has a lower polymer chain mobility than the bulk polymer, the resultant denser structure and the reduced free volume decreases permeation flux and increases selectivity [28,55]. Le et al. [35] developed PEBA/polyhedral oligosilsesquioxane (POSS) membranes. They reported that the addition of a small amount of POSS nanoparticles could disrupt the inherent organization of polymer chains. This would enhance the available free volume in the polymer matrix by reduction of polymer strength. However, it was also demonstrated that increased loading of the nanoparticle constrained movement of polymer chains through the hydrogen bonding interaction between oxygen atoms on PEBA polymer chains and hydroxyl groups on POSS molecules which in turn lowered the free volume in the polymer matrix [35]. Adoor et al. [13] developed nanocomposite membranes composed of PTA nanoparticles incorporated into SA polymer. The characterization of resultant membranes revealed that that the nanoparticles occupy the free void spaces of the polymer and plasticization of the polymer was reduced. In a study carried out by Amirilargani et al. [27], PSS-wrapped MWCNTs were incorporated into the PVA matrix to prepare nanocomposite membranes. It was reported that wrapping MWCNTs with PSS leads to good affinity between the polymer matrix and MWCNT particles and there was no sign of interfacial voids in morphology of the resultant membranes. Their results revealed that low content of MWCNTs reduced membrane free volume and made polymer matrix more rigid due to reduced polymer chain mobility as well as good interaction between PVA and MWCNTs. However, it was found that at higher additive loadings, membrane free volume increased due to agglomeration in the polymer matrix. Panahian et al. [49] investigated modified-MWCNTs/PVA nanocomposite membranes and showed that nanofillers acted as reinforcing bridge elements in the membrane. This caused a reduction in the free spaces of the polymeric matrix such that rigidity of the polymeric chains increased. However, it was reported that at higher nanofiller content in the membrane, the free volume increased due to agglomeration of CNTs in the membrane matrix [49]. Salehian et al. [22] studied the free volume properties of iron (III) acetylacetonate (FeAc)/PI membranes by the PALS technique. The results showed that fractional free volume decreased after incorporation of FeAc into the membranes. The same result was reported by Penkova et al. [50] in their study on nanocomposite membranes of PPO and fullerene. Julluk et al. [72] studied the effect of incorporation of modified SiO_2_ nanoparticles into polyphenylsulfone (PPSU) polymer matrix and the results indicated a decrease in porosity of the membrane and formation of denser membranes. Similarly, in another study carried out by Penkova et al. [51], it was found that addition of fullerenol to the PVA decreased free volume inside the polymer film and the resultant membrane became more rigid. Khoonsap et al. [104] used the PALS technique to study free volume characteristics of SiO_2_ nanosphere/PVA nanocomposite membranes. It may be concluded that free volume size decreased due to interactions between SiO_2_ nanospheres and PVA chains. Similarly, Nigiz et al. [124] found that addition of POSS filler into PEBA membrane reduced flexible chain mobility and free chain spaces of polymeric matrix. In a study done by Garg et al. [32], it was reported that in PDMS/clay nanocomposite membranes, the molecular dynamics of polymer chains were restricted by addition of nano-clay. Investigating N-p-carboxy benzyl chitosan (NCBC)-sulfonated silica-PVA nanocomposite membranes for PV dehydration of ethanol, Tripathi et al. [87] found that increasing the NCBC-silica content in the membrane matrix reduces its void porosity. Ong et al. [48] aligned PHB-functionalized MWCNTs into a CS matrix to prepare nanocomposite membranes for PV dehydration. The resultant membrane morphology was compared to that of the PHB membrane and raw MWCNT/CS nanocomposite membrane to determine the compatibility of the PHB-MWCNT/CS nanocomposite membrane. They found that the PHB-MWCNT was completely incorporated into the CS layer rather than simply covering the surface. This indicated that the interfacial adhesion between the MWCNTs and the polymer matrix was enhanced due to functionalization of MWCNTs with PHB [48]. Similarly, Yeang et al. [55] investigated the PV performance of nanocomposite membranes comprising PVA functionalized MWCNTs and CS and found that flexibility of polymer chains as well as free volume of the polymer matrix decreased by addition of PVA-MWCNTs [55]. Sun et al. [82] investigated organophilic nano-silica (ONS) filled PDMS composite membranes reported that even though the silica nanoparticles were only physically blended in the PDMS polymer matrix, a dense structure with no apparent voids at the interface of the PDMS and nano silica particles were obtained.

#### 4.1.2. Membrane Crystallinity

Presence of nanoparticles in polymeric nanocomposites does not only change free volume but it also affects polymer crystallinity in terms of change in chain diffusion and nucleation. It has been reported that if nanoparticles disturb polymer chain mobility in such a way that the diffusion process of the polymer chains is hindered, crystallinity will decrease. In contrast, crystallinity will increase if nanoparticles serve as the nuclei and increase the nucleation rate of the crystallization process [100].

Several studies revealed that incorporation of nanoparticles into a polymer matrix can potentially reduce polymer crystallinity [56]. It is generally agreed consent that the mass transfer properties of membrane are significantly affected by the crystalline phase of polymer and generally mass transfer is higher in the polymer with low crystallinity [56]. In a study done by Yang et al. [90] on CS/TiO_2_ nanocomposite PV membranes, it was reported that incorporation of TiO_2_ nanoparticles into CS structure led to a reduction in crystallinity of membranes, and increase flexibility of polymer chains [90]. Mandal et al. [76] indicated that the presence of iron oxide in the PVA polymer matrix increased the amorphous characteristics of the nanocomposite membranes. A similar reduction in crystallinity was observed in a study carried out by Lin et al. [75]. Investigating nano SiO_2_/PVA composite membranes, the results demonstrated that nano silica disrupted crystallization of PVA in such a way that amorphous regions and free volume increased [75]. In a more recent work done by Gao et al. [56], PV nanocomposite membranes were fabricated by incorporating MWCNT, which was decorated by magnetite (Fe_3_O_4_) nanoparticles, into SA. It was reported that attachment of Fe_3_O_4_ nanoparticles to the MWCNT surface could prevent their aggregation in the membrane matrix.

Furthermore, the authors argued that crystallinity of the polymer decreased due to the high interaction between polymer and additive. Therefore, the amorphous regions, as well as free volume voids in the membrane matrix, increased. As a result, they concluded that the increased free volume in the polymer matrix or at the interface between the polymer matrix and nanoparticles could facilitate diffusion of penetrant molecules through the membrane [56]. In a more recent study Torabi et al. [86] synthesized PVA/SiO_2_ nanocomposite membranes for PV dehydration. Membrane characterization revealed that silica nanoparticles restricted formation of crystals such that the crystallinity of nanocomposites was reduced and accordingly amorphous regions developed in the polymer [86]. Liu et al. [106] fabricated PVA hybrid membranes and showed that Fe-DA nanoparticles destroyed crystalline areas of the PVA matrix causing increased fractional free volume in the PVA matrix. Investigating the properties of PV nanocomposite organoclay/poly (styrene-co-butyl acrylate) membranes, Samanta et al. [95] found that the crystalline structure of the clay was broken in the filled membrane because of strong electrostatic interaction among the functional groups of clay and copolymer.

### 4.2. Swelling Degree

As mentioned earlier, the transport through PV membranes is generally described by the solution-diffusion mechanism including three consecutive steps in which sorption of the permeation species from the feed mixture to the membrane surface is a critical step [35]. Sorption is strongly related to membrane affinity with permeants and it is affected by molecule characteristics and membrane properties [35]. Generally, the swelling degree of a membrane can quantitatively describe membrane affinity for the feed mixture [76].

The membrane swelling is a significant factor particularly in the PV process since it affects permeation of feed components through the membrane during the separation process [3,86]. In fact, interactions between the membrane and permeants molecules may swell the membrane structure which leads to a loose polymer chain packing [35]. In detail, when the membrane swells, polymer chains are stretched and free volume in the membrane structure increases which leads to an increase in flux and decrease in membrane selectivity [32,52,81].

Membrane swelling in certain liquids can be affected by several parameters such as chemical compositions, physicochemical properties, and structure of the membranes [3]. In nanocomposite membranes, the combination of nanoparticles with polymer can give rise to two outcomes in membrane structure which leads to two different swelling behavior: (i) nanoparticles restrain mobility of polymer chains and introduce non-swelling regions in the polymer matrix in such a way that the swelling degree of membrane reduces [32,41,55,87] and (ii) nanoparticles increase free volume in the membrane matrix due to interfacial gaps between nanoparticles and polymer matrix and accordingly increase swelling degree of membrane [62,97].

Beltran et al. [41] investigated characteristics of SiO_2_/PDMS membranes for pervaporative recovery of 1-butanol from aqueous solution. Gravimetric analysis was used to determine the swelling degree of the membranes. It was reported that the nanocomposite membranes showed lower values of swelling degree compared to pure PDMS membrane. This result was attributed to the effects of SiO_2_ particles on the membrane structure by introducing non-swelling regions in the polymer matrix as well as reducing polymer chain mobility. However, a contradictory result was presented by Sun et al. [82] investigating organophilic nano-silica filled PDMS composite membranes for pervaporative separation of ethanol/water mixture. They reported an enhancement of swelling degree of nanocomposite membranes since incorporation of nanoparticles could restrict the tight packing of PDMS chains and lead to an increase in accessible free volume in the polymer matrix.

The reported changes in the swelling degree of PV membranes after incorporation of nanofillers are summarized in Table 6.

### 4.3. Water Contact Angle Parameter

Contact angle is a parameter to indicate wetting properties of the membrane relative to the strength of the interfacial interaction between the feed mixture and the membrane [43]. In PV, both hydrophilic and hydrophobic membranes can be used for separation depending on the application [15]. In order to characterize the surface hydrophilicity or hydrophobicity of the membranes, contact angle analysis can be used in which the angle formed by a water droplet and the membrane surface is measured [15,94]. In general, if the water contact angle is higher than 90°, the membrane surface is considered hydrophobic. Otherwise (water contact angle lower than 90°) membrane surface is considered hydrophilic [15,43]. As a matter of fact, the contact angle is an important parameter to identify the degree of wettability of a membrane surface, which mainly depends on membrane surface chemistry as well as the surface roughness [10,15,140]. In other words, the membrane properties can be significantly affected by its surface texture. It should be noted that as the surface roughness increases, the effective surface area increases and this could affect the wettability of materials such that a hydrophobic surface becomes more hydrophobic and a hydrophilic surface more hydrophilic depending on the nature of surface materials (thus the membrane has a chance to promote the permeation rate) [10,12,141].

From the contact angle analysis of functionalized MWCNT/PVA nanocomposite membranes, Jose et al. [47] found that hydrophilicity increased in the PVA matrix because the surface of nanocomposite contains acid functional groups which decrease the hydrophobic nature of PVA membranes.

Reduction in the contact angle values of zeolite/PA nanocomposite membranes was reported by Fathizadeh et al. [10]. It was found that the hydrophilicity of the PA thin film layer increased because the surface roughness increased the effective surface area [10].

Water contact angle results for the PV nanocomposite membranes are reported in Table 6. As can be seen, few studies have attempted to investigate the effects of nanoparticles on the water contact angle of nanocomposite membrane.

## 5. The PV Separation Performance of Nanocomposite Membranes

The PV performance of membranes can be evaluated by two sets of interlinked parameters of a membrane. First set: flux and separation factor, and second set: permeability (or permeance) and selectivity [142].

In the first set, the flux of a membrane (*J*) can be directly determined from the experiment by evaluating the total weight of permeate (*Q*) collected at a specific period (*t*) over the effective surface area (*A*) of the membrane [8]:

(1) $$J=\frac{Q}{A\times t}$$

On the other hand, permeability (*P*) is the ability of permeates to pass through a membrane which can be determined by the following equation [8,143]:

(2) $$J_{i}=\frac{P_{i}}{l}\left({\left(x_{i}\gamma_{i}p_{i}^{sat}\right)}_{f}-{\left(x_{i}\gamma_{i}p_{i}^{sat}\right)}_{p}\right)$$

where *P_i_* is permeability of component *i* across the membrane, *l* is thickness of the selective layer, *xi*, *γi*, and *p_i_^sat^* are mass fraction, activity coefficient and saturated vapor pressure of component *i*, and subscripts *f* and *p* denote the feed and permeate sides of the membrane, respectively [8].

The second set of parameters can be used to evaluate separation efficiency of the membrane using the term separation factor (*β*) or selectivity (*α*). The separation factor can be expressed as follows [6,20]:

(3) $$\beta =\frac{C_{i,p}}{C_{i,f}}$$

where *C* is the concentration of selectively permeating solute.

The other parameter, selectivity, which for a binary feed mixture can be defined as the ratio of permeability of component *i* to the component *j* [8,20,143,144]:

(4) $$\alpha_{ij}=\frac{P_{i}}{P_{j}}$$

Generally, a trade-off between permeation flux and selectivity exists such that an increase in selectivity of a membrane leads to a decrease in permeability and vice versa [20]. There is a practically combined parameter, which is called PV separation index (PSI), to determine separation ability of a membrane [20]. This parameter is defined as follows [20,44]:

(5) PSI=(α−1)×J

The “trade-off” between selectivity and permeability in most integral membranes has led to the development of nanocomposite membranes [49]. Up to now, assorted studies have been carried out to establish nanocomposite membranes with higher PV separation performance as compared to pure polymeric and inorganic membranes. In general, studies on PV performance of nanocomposite membranes can be divided into three categories based on their applications whose experimental results and arguments of related investigations are discussed in the following parts.

### 5.1. Dehydration of Aqueous-Organic Mixtures by Hydrophilic Nanocomposite Membranes

For dehydration of organic solvents containing small amounts of water, PV membranes preferentially allow water to permeate through because of their higher affinity for water than organic solvent [7,8,10]. In this case, hydrophilic membranes such as PVA, polyacrylonitrile (PAN), PA, PI, CS, PES, and alginate are often used to remove water [11,20,42,48,145]. However, most of the present hydrophilic membranes used for PV dehydration can swell easily in the presence of water during operation which might degrade membrane structure and affect separation performance [12,22,96]. In fact, when the membrane swells, polymer chains are stretched which leads to increased flux but decreased selectivity of membranes. Moreover, some of the PV membranes have thermal and chemical stability issues at some feed concentrations and temperatures [12,66]. To deal with these problems, incorporating hydrophilic nanofillers in the matrix of polymers is an effective modification method to prepare new chemically and thermally stable membranes with high separation performance [8,10,96].

Up to the present time, most studies on hydrophilic PV nanocomposite membranes have been based on the use of nanofillers such as Fe_3_O_4_, TiO_2_, SiO_2_, CNTs, and zeolites (as listed in Table 1). Table 7 compiles experimental results of studies on PV performance of nanocomposite membranes for dehydration of aqueous-organic mixtures.

The studies performed on nanocomposite membranes containing these nanofillers are summarized in the following while being categorized based on the utilized polymer.

#### 5.1.1. PVA Nanocomposite Membranes

Among the hydrophilic membranes which have been investigated for PV dehydration process, PVA is the most studied one due to its high hydrophilicity, good chemical and thermal stability, and outstanding membrane-forming ability with excellent water perm-selective properties [16,27,42,47,62,86,94,145]. In spite of their potential advantages, PVA membranes suffer from the drawback of high swelling in aqueous solutions due to presence of hydrophilic groups in the polymer which leads to a decrease in membrane selectivity and stability [16,27,47,51,53,86,94,97]. Therefore, some modification methods have been developed to overcome swelling and enhance performance of these membranes [42,48,94,97]. According to the literature, it seems that recent trends have shifted towards incorporation of nano-sized particles in the polymer matrix and a variety of nanofillers such as CNTs [27,42,47,49,53,101], nanostructured ZIF [62], nano-sized PANI particles [97], cellulose nanocrystals [31], nanoclay [94,102], SiO_2_ [45,73,75,86,100], TiO_2_ [71], and Fe_3_O_4_ nanoparticles [76,81] were incorporated into PVA membranes to prepare nanocomposites using PVA as base polymer.

Incorporation of CNTs has attracted considerable interest due to their extraordinary mechanical, electrical, thermal, and more importantly, excellent separation properties [42,55]. However, preparation of homogeneous nanocomposite membranes with CNTs is difficult because of their tendency to agglomerate [42,48,55]. Hence, there is a requirement for functionalization of CNTs with desired functional groups to have better dispersion efficiency [42,43,55]. Several studies have been carried out to investigate the potential of utilizing CNTs in preparing nanocomposite PVA/CNTs membranes for PV process. For instance, in an experimental study performed by Peng et al. [34] novel nanocomposite membranes composed of CS-wrapped MWCNTs and PVA were prepared for pervaporative separation of benzene and cyclohexane mixtures. Their results demonstrated that in comparison with pure PVA membrane, nanocomposite PV membranes exhibit simultaneous increase of permeation flux and separation factor [34]. In another study carried out by Shirazi et al. [42,53], highly pure and functionalized CNTs were incorporated into PVA to prepare PVA-CNTs nanocomposite membranes for dehydration of IPA. In this study, CNTs were synthesized by the CVD method and then purified and functionalized by nitric acid. The results revealed that presence of CNTs in the PVA matrix considerably reduces swelling and increases water selectivity. These improvements have been attributed to the fact that the incorporation of CNTs compacts and rigidifies PVA matrix and decrease its free volume. However, it was reported that the permeation of water molecules through nanocomposite membranes decreases due to the polymer chains rigidification [42,53]. In another research study done by Amirilargani et al. [27], nanocomposite PV membranes composed of PVA polymer and functionalized MWCNTs were prepared for dehydration of IPA. In their study, PSS was used as a functionalization agent for wrapping MWCNTs to improve nanotubes dispersion in the polymer [27]. It has been reported that addition of MWCNTs-PSS to PVA matrix improved membrane performance such that membranes have significant ability to selectively separate water from water-IPA mixtures [27]. Recently, Jose et al. [47] prepared PVA nanocomposite membranes with acid functionalized MWCNTs and used them to separate azeotropic composition of water-ethanol mixtures. The PV performance of nanocomposite membranes was studied in terms of permeance and selectivity. The experimental results showed an increment in intrinsic selectivity. They concluded that separation efficiency is influenced by morphology and hydrophilic nature of the membranes and interaction of filler and permeant [47].

Apart from CNTs, other types of nanomaterials such as metal oxides have been used to prepare PVA nanocomposite membranes for PV separation. For example, Sabetghadam et al. [80] prepared nanocomposite membranes composed of PVA and silica nanoparticles using in situ sol-gel method for PV separation of ethanol/water mixtures. In detail, PVA as a continuous phase in nanocomposite membranes was cross-linked by two types of silanes coupling agents through co-hydrolyzation and co-condensation reactions during the sol-gel process to create nanoparticles in the polymer matrix. An improvement was observed incompatibility between the polymer matrix and silica nanoparticles in the nanocomposite membranes. The results of this study indicated that condensation reaction of hydroxyl groups of PVA and alkoxysilane leads to restricting polymer chains movement such that the main reason reported for improvement of separation performance of membranes was crosslinking with alkoxysilane.

Similarly, Lin et al. [75] investigated synthesizing nano silica/PVA composite membranes onto PAN support and used them for PV dehydration of water/caprolactam (CPL) mixture. Compared to the pure PVA membranes, nanocomposite membranes showed good performance in PV separation process in terms of increased permeation flux due to membrane’s hydrophilicity enhancement in the presence of silica nanoparticles. Torabi et al. [86] synthesized cross-linked PVA/silica nanocomposite membranes and used them for dehydration of methyl acetate reaction mixtures by PV process. Based on their results, incorporation of silica nanoparticles in the polymer chain enhanced the number of polar OH groups in the polymer matrix which led to an increase in water permeation. Furthermore, it was found that diffusion of other components with large molecular sizes decreased because of the obstructions made by silica nanoparticles in nanocomposite membranes [86].

Mandal et al. [76] attempted to improve the dimensional stability of PVA membrane using iron oxide nanoparticles to enhance its PV performance for dehydration of water-acetonitrile. The resultant nanocomposite membranes showed improved flux and selectivity [76]. Bai et al. [31] investigated the effect of cellulose nanocrystals in PVA/cellulose nanocomposite membranes on PV performance for ethanol dehydration. It has been found that incorporation of cellulose nanocrystals leads to significantly increased separation factor, whereas the membrane flux slightly decreased [31]. Naidu et al. [97] prepared nanocomposite membranes containing nano-sized PANI particles dispersed in PVA and used them in PV separation of water-IPA mixtures. It was observed that flux of nanocomposite membranes decreased in comparison with pure PVA membrane. However, the selectivity enhanced significantly [97].

Furthermore, the flux increased with increasing concentration of PANI nanoparticles in the PVA matrix due to higher hydrophilic-hydrophilic interactions. Moreover, selectivity increased due to reduced membrane swelling [97]. In another study, Aminabhavi et al. [71] examined PV separation performance of PVA nanocomposite membranes filled with PANI-coated TiO_2_ and TiO_2_ nanoparticles for dehydration of aqueous mixtures of 1,4-dioxane and tetrahydrofuran. Their results showed that nanocomposite membrane selectivity for water increased as compared to unfilled cross-linked PVA membrane because TiO_2_ particles made PVA chains tighter.

On the other hand, incorporation of surface-modified PANI-coated TiO_2_ nanoparticles in the PVA matrix led to higher flux [71]. Amirilargani et al. [62] investigated PV properties of PVA/ZIF membranes for IPA dehydration. It was found that addition of ZIF to PVA matrix led to an increase in permeation flux along with decreased separation factor [62]. This is primarily because of interfacial gaps between ZIF nanoparticles and PVA matrix [62]. To investigate the overall performance of membranes, PSI values of PVA/ZIF membranes were calculated. The results showed an increase in PSI by increasing nanoparticle loading up to an optimum concentration, and then PSI decreased by further increase of ZIF concentration because of sharp reduction of separation factor which was caused by agglomeration of ZIF nanoparticles in the PVA matrix [62].

#### 5.1.2. CS Nanocomposite Membranes

In the field of hydrophilic nanocomposite membranes, materials other than PVA have also been studied for dehydration of aqueous-organic mixtures by PV. For instance, CS is a polymer which has long been studied for dehydration by PV process due to its remarkable water permselectivity for various water/organic solvent mixtures, good durability to organic solvents and presence of functional groups (amine and hydroxyl groups [24]) which can be easily modified [16,145]. Incorporation of nanoparticles such as CNTs [48,54,55], titanate nanotubes (TNTs) [44], Fe_3_O_4_ [4], and titanosilicate [21] into this polymer to form a nanocomposite membrane has the potential for further improvements of membrane performance.

Sudhakar et al. [54] investigated PV performance of CS nanocomposite membrane containing MWCNTs for separation of IPA/water azeotropic mixture. The membranes exhibited high permselectivity towards water for all feed compositions studied [54]. Simultaneous increase in flux and separation factor was due to packing of hydrophilic chains in the matrix as well as internal nanochannels provided by MWCNTs. In an interesting work done by Ong et al. [48], PHB-functionalized MWCNTs/CS nanocomposite membranes were synthesized and used in dehydration of 1,4-dioxane. In detail, the MWCNTs were first functionalized with PHB to improve their compatibility with CS and then aligned into the CS matrix [48]. It was found that excessive swelling behavior of CS had decreased due to the incorporation of PHB-MWCNTs so that the resultant nanocomposite membranes had a relatively high water permeation flux and selectivity [48]. Yeang et al. [55] investigated PV performance of functionalized MWCNTs/CS nanocomposite membranes in dehydration of acetone. In order to improve compatibility and dispersion of MWCNTs in the CS matrix, they were functionalized with PVA as a hydrophilic, non-toxic and degradable synthetic polymer [55]. It was found that at increased PVA-MWCNT loading, in spite of lower free volume, higher water permeability was achieved with a slight selectivity reduction [55]. Dudek et al. [4] investigated PV separation performance of water/ethanol mixture by nanocomposite CS membranes filled with various amount of Fe_3_O_4_ and discussed the influence of iron oxide on transport properties. It was reported that after addition of iron oxide nanoparticles to the polymer matrix, water permeation gradually increased, while ethanol permeation decreased. Yang et al. [90] studied separation performance of ethanol-water mixture as a model system of CS/TiO_2_ nanocomposite membranes by PV process. It was reported that permeation flux of nanocomposite membranes increased with increasing TiO_2_ content up to an optimum concentration. They argued possible reasons for this change in transport properties of CS membrane: increase in amorphous region in the CS matrix caused by formation of TiO_2_ particles in the CS matrix, and hydrophilicity change of nanocomposite membranes. It was reported that for all hybrid membranes, the diffusion selectivity was smaller compared to pure CS membrane. The reason of this phenomenon could be attributed to the presence of nanovoids which formed by the incorporation of inorganic fillers. Furthermore, it should be noted that too high nanoparticle loading affected the swelling degree of these hybrid membranes. They found that all of hybrid membranes exhibited higher swelling degree than pure CS membrane. This may be due to the fact that by incorporation of TiO_2_ particles, the flexibility of CS chains increased, as well as the interaction between water molecules and TiO_2_ particles in the hybrid membranes. TNTs/CS nanocomposite membranes with high PV performance for IPA dehydration were prepared by Liu et al. [44]. The effect of TNTs content on performance of the membrane for separation was investigated. It was found that after incorporation of nanotubes, both flux and separation factor increased. They discussed that this change was basically due to efficient size-selective channels formed by nanotubes due to their tubular structure, and also their high hydrophilicity and large surface area. Casado et al. [21] prepared titanosilicate/CS nanocomposite membranes and used them in dehydration of ethanol by PV. The nanocomposite membranes showed higher PV flux but lower water/ethanol separation factor compared to CS membrane [21]. According to literature, the incorporation of inorganic fillers into the CS matrix usually decreases hydrophilicity of the resultant membrane. However, in this study, a negligible change in hydrophilicity was observed [21].

Having mentioned the effect of nanofillers on CS nanocomposites, it should be noted that most of the studies have shown limited increase in flux which could be attributed to factors such as (i) low loadings of inorganic fillers in the polymer matrix, (ii) chain rigidification and partial pore blocking, and (iii) low interfacial areas between polymer and porous fillers because of large inorganic fillers [43,94].

#### 5.1.3. PI and PA Nanocomposite Membranes

Another valid candidate for preparation of membranes useful in dehydration is PI and PA which present excellent mechanical, thermal, chemical resistance, and reasonable solvent resistance [16,145]. However, compared to most conventional polymeric materials, PI membranes swell in aqueous solutions which might degrade membrane structure and separation performance [22]. In order to obtain membranes with high separation performance, incorporating nano-sized zeolite [7], nanostructured ZIF [63], MgO [3], and iron oxide [22] nanoparticles in PI have been studied.

Bakhtiari et al. [7] investigated PV performance of zeolite/PI membranes for pervaporative dehydration of isopropanol/water mixtures. The results indicated that incorporation of hydrophile zeolite fillers into the PI matrix improved its separation performance in terms of higher flux and higher selectivity. Salehian et al. [22] fabricated PI composite membranes with an adjusted pore size and pore distribution and used them in the PV process for dehydration of IPA/water mixtures. As a result, these membranes showed superior performance in terms of high permeability and selectivity for this application. It was noted that presence of iron oxide nanoparticles in nanocomposite membranes increased water sorption uptake. Jiang et al. [3] studied the dehydration of isopropanol by PV using MgO/PI membranes. They reported that co-existence of hydrophilic channels, formed by carboxylic groups, and hydrophobic channels, created by hydrophobic parts of the polymer matrix, could lead to higher selectivity and lower permeability, respectively.

Similarly, Hua et al. [63] investigated PV performance using ZIF/PI membranes for isopropanol dehydration. It was found that at low ZIF loading, incorporation of ZIF into PI efficiently increased permeate flux while separation factor was similar to the pristine PI membrane. By increasing ZIF loading in the nanocomposite membranes, the separation factor decreased due to presence of void volume. Sokolova et al. [117] studied PV transport properties of PI/ZrO_2_ nanostars membranes for separation of butanol-water mixture. Addition of ZrO_2_ nanostars to semi-crystalline PI led to increased selectivity due to formation of water-selective channels along organic-inorganic interface.

Similarly, studies to modify PA membranes using hydrophilic nanofillers such as nano-sized zeolite and nano clay in the polymer matrix resulted in nanocomposite membranes with improved performance in dehydration by PV [10,37]. Wang et al. [37] prepared PA/sodium dodecyl sulfate (SDS)–clay nanocomposite membranes and utilized them in PV dehydration of aqueous ethanol mixtures. Compared to pure PA membranes, higher separation factor was observed for nanocomposite membranes. Fathizadeh et al. [10] studied pervaporative dehydration of aqueous alcohol solutions using PA/nano zeolite thin film nanocomposite membranes. Their results indicated an improvement in separation performance by introduction of nano NaX zeolite particles in the PA active layer. In details, they reported that water flux and separation factor of these membranes was about twice that of pure PA membrane.

#### 5.1.4. SA Nanocomposite Membranes

SA is also considered as an excellent candidate in membrane preparation for dehydration due to promising properties such as high hydrophilicity and good film formation characteristics [16,20,145]. Though, because of its low mechanical and thermal stability and high solubility in water, some modifications are required to improve life of membrane and reduce degree of swelling [16]. In this regard, incorporating nanoparticles such as CNTs [56] and TiO_2_ [36] in SA polymer have been studied to improve its separation performance. Gao et al. [56] evaluated PV dehydration performance of MWCNT/SA membranes for ethanol-water mixtures. In their study, MWCNTs were decorated by hydrophilic Fe_3_O_4_ nanoparticles to lower their surface tension which can effectively prevent their aggregation. The results showed that both permeation flux and separation factor of composite membranes were higher than that of pure membranes due to formation of fast-moving micro-channels.

Furthermore, it has been reported that with increasing content of fillers, separation factor of composite membranes decreases due to reduction in the membrane hydrophilicity. In a study carried out by Lokesh et al. [36], dehydration of 1,4-dioxane/water mixtures by PV process using PANI-coated titanium dioxide/SA nanocomposite membranes was investigated. Their results indicate that selectivity of nanocomposite membranes increases while flux decreases when compared to unfilled membranes. This is due to the presence of fillers which act as the reinforcing bridge elements in the membrane matrix so that polymer chains become tighter. In another study, Zhao et al. [111] investigated the effect of incorporating zwitterionic GO into SA membrane for dehydration of water/alcohol mixture. PV results showed higher separation performance of these membranes compared to SA and unmodified GO/SA membranes, attributing to continuous pathways provided by GO as well as high-density zwitterionic groups which conferred electrostatic interaction sites with water molecules. In a study done by Xing et al. [113], it was found that permeation and selectivity of SA membrane for ethanol dehydration by PV were enhanced by incorporating attapulgite nanorods due to their selective channels and hydrophilic groups. Adoor et al. [13] studied dehydration of isopropanol and ethanol using PV nanocomposite membranes composed of phosphotungstic acid (PTA) nanoparticles as fillers in SA. Compared to pure membranes, the separation performance of nanocomposite membranes was improved in terms of simultaneous enhancement of enrichment factor and PSI. Moreover, PTA nanoparticles were chemically modified using ammonium carbonate to exchange excess protons with larger (more abundant) NH_4_^+^ ions in order to reduce leaching of filler particles. In a recent work, Wang et al. [112] investigated PV performance of nanocomposite membranes comprising of SA polymer and poly (ethylene glycol)-functionalized polyoctahedral oligomeric silsesquioxanes (PEG@POSS) nanoparticles for dehydration of ethanol/water solution. This PEG modification was used to enhance the permeation of water molecules into the membrane and thus the selectivity of the membrane as well as permeate flux increased.

### 5.2. Removal of Organics from Aqueous-Organic Mixtures by Hydrophobic Nanocomposite Membranes

Hydrophobic membranes such as PDMS [26,41,59,61,68,79,82,99,121,122], PEBA [12,35,107,123] and PVDF [52] are used to selectively remove traces of organic compounds from an aqueous solution by PV. Studies on PV performance of nanocomposite membranes in this application are presented in Table 8.

In this case, the hydrophobic nature of membranes allows organics to transport which limits permeation of water [16]. However, in comparison with organic solvents, water has a lower molecular size and it can diffuse easily through the membrane leading to a reduction in membrane performance [16].

The most common polymer employed for preparation of hydrophobic PV membranes is PDMS, often referred to as “silicone rubber” [16,58,69]. It has some advantages such as excellent film-forming ability, good thermal and chemical stability and hydrophobicity. However, its flexible macromolecular chains facilitate diffusion of all components through the free volume of the polymer matrix which causes low selectivity of these membranes [16,32,58,60,69]. Several methods have been adopted for modification of PV performance of PDMS membranes [32]. A simple and widely accepted method to improve their separation performance is incorporation of nanoparticles such as silicalite [58,59,60,61], silica [26,69,79,82,92,120], PZSNT [99] and ZIFs [67,68,69,121] into the polymer matrix at prepolymer stage to prepare nanocomposite membranes.

Lu et al. [59] used silicalite/PDMS nanocomposite membranes for acetic acid/water separation by PV process. As compared to plain PDMS membranes, an improvement on both separation factor and permeation flux were achieved because of higher sorption selectivity of nano-sized silicalite particles for acetic acid. Liu et al. [58] fabricated ultrathin homogeneous silicalite/PDMS nanocomposite membrane on capillary support for pervaporative recovery of iso-butanol from aqueous solution. These nanocomposite membranes exhibited very high flux and good separation factor which was attributed to hydrophobic channels of silicalite nanocrystals and thus selective permeation of iso-butanol molecules. Yadav et al. [61] used silicalite/PDMS nanocomposite membranes for ethanol separation from dilute aqueous solutions by PV. It was found that both alcohol flux and separation factor increased by increasing nano silicalite loading in nanocomposite membranes which attributed to the larger surface area provided by silicalite nanoparticles.

Tang et al. [85] investigated PV performance of fumed-silica-filled PDMS–PA composite membranes for separation of ethanol from aqueous solution. Addition of fumed silica into polymer matrix led to an increase in ethanol permeation through the membrane which could be attributed to disruption of PDMS chain packing by introduction of fumed silica. Furthermore, the selectivity did not decline significantly due to hydrophobic features of fumed silica. Similarly, Peng et al. [79] utilized silica/PDMS nanocomposite membranes for ethanol recovery by PV. They reported that introduction of silica particles modified with a silane coupling reagent significantly led to improved PV performance of composite membranes. This result was attributed to disruption of polymer chain packing by silica nanoparticles as well as an increase in membrane free volume. Shirazi et al. [26] studied the application of silica/PDMS nanocomposite membranes for recovery of isopropanol from aqueous solution by PV. Based on their results, it was found that by introducing silica nanoparticles into the membrane, selectivity significantly increased, and permeation flux decreased because of rigidification of polymer chains. In a study presented by Sun et al. [82], PV separation of ethanol/water mixture by organophilic nano-silica/PDMS nanocomposite membranes was investigated. Their results indicated that with increasing nano-silica concentration in PDMS matrix, solubility selectivity and diffusion selectivity of the resultant membrane increased due to organophilic properties of nano-silica. However, with a further increase of nanoparticles content in the PDMS membrane, the total flux decreased because water sorption and diffusion in the filled membranes decreased. Beltran et al. [41] functionalized the surface of silica nanoparticles before using them as fillers in the PDMS membrane for pervaporative recovery of 1-butanol from aqueous solution. In their study, functionalizations were carried out using three different chlorosilanes as silylating agents to reduce hydrophobicity of fumed SiO_2_ surface and it was found that the resultant membranes exhibited better separation performance. In a study carried out by Nourani et al. [60], the effects of silica and silicalite nanoparticles as fillers in the PDMS matrix on pervaporative performance of membranes for purification of toluene from dilute aqueous solution were investigated. The results showed that by incorporating fillers into PDMS, enrichment factor of the composite membrane significantly increased. It was reported that the fillers could restrain mobility of polymer chains by filling the spaces between them which caused decreased permeation flux. However, a reverse trend was observed at higher filler loadings due to the formation of defects and interfacial voids in the membrane structure. Zhou et al. [92] fabricated SiO_2_/PDMS nanocomposite membranes the separation of ethanol from an aqueous solution by PV. Their result indicated that by using these membranes, the separation factor significantly increased and a higher total flux was observed even at a high feed flow rate which confirmed that the membranes could overcome the trade-off phenomenon in the PV process.

In a study for ethanol separation from aqueous solution by PV, Huang et al. [99] prepared PZSNTs/PDMS nanocomposite membranes. They reported that the resultant membranes showed a higher separation factor in comparison with pure PDMS membranes. They also noted that by increasing the content of PZSNTs, both selectivity and permeation flux increased at first to a maximum value and then reached a plateau.

Shams et al. [120] investigated pervaporative removal of toluene from water by silica/PDMS membranes in which particles were dispersed in the active layer using a mediating surfactant. It was found that both permeation flux and selectivity factor of the membrane for toluene increased owing to enhancement in hydrophobic character of composite membranes. Wang et al. [68] investigated recovery of butanol from aqueous solution by PV process using ZIF/PDMS membranes. The results showed an enhanced total flux due to the enlarged free volume in the polymer matrix, and higher separation factor compared to pure PDMS membrane due to super-hydrophobic ZIF pore channels which provided a more organophilic contact surface for butanol to pass through the membranes.

Yin et al. [121] prepared ZIF/PDMS nanocomposite membranes and investigated their PV performance for recovery of ethanol and 1-butanol from aqueous solutions. It was found that with increasing ZIF loading, membranes showed an increase in both ethanol and water permeability as a result of the loose chain packing of the PDMS polymer matrix. However, selectivity of membranes increased due to the hydrophobic nature of ZIF particles. In a study done by Azimi et al. [122], the effect of activated carbon nanoparticles on the PV performance of PDMS membrane was investigated for separation of butanol from aqueous solution. They reported that compared to neat membranes, an increase in both flux and separation factor was observed which attributed to the porous structure of the nanofillers. It was noted that this porous structure provided new pathways as well as additional sorption sites for facilitated mass transport through the membrane.

PEBA is another polymer which has been studied for removal of organic compounds from aqueous solutions by PV [12,35,123]. This polymer is a block copolymer material comprising hard PA segments which promote mechanical strength and soft polyether (PE) segments which provide good affinity to organic solvents [35]. In order to improve permeability and selectivity of PEBA, addition of nanoparticles into the polymer could be helpful [146]. Few studies are available on PEBA nanocomposite membranes used for removal of organics from aqueous-organic mixtures by PV [12,35,123,146].

For instance, Le et al. [35] investigated separation performance of POSS/PEBA membranes for ethanol recovery by PV process. In their study, two types of POSS particles, octa (3-hydroxy-3-methylbutyldimethylsiloxy) and disilanolisobutyl, were incorporated into the PEBA matrix. It was reported that the total flux also increased because POSS particles facilitated ethanol transport through the membranes by opening up micro-voids at the particle–polymer interface. Moreover, the results showed an enhancement in the separation factor of the membranes which was attributed to the induced preferential sorption of membranes towards ethanol by POSS particles. Similarly, in a study done by Nigiz et al. [124], aminopropyl isobutyl POSS/PEBA membranes were fabricated for n-butanol recovery from its aqueous solution. It was reported that by increasing POSS loading in the membrane up to a specific value (3 wt.%), a simultaneous enhancement of flux and separation factor is observed due to the hydrophobic nature of POSS particles. However, at higher content of fillers, the separation factor increased while a decrement in flux was observed which was attributed to the fact that the filling-blocking effect of POSS particles could overcome their hydrophobic properties.

Sunitha et al. [12] studied the PV performance of zeolite/PEBA nanocomposite membranes for dehydration of NMP/water mixtures. It was found that the presence of hydrophilic zeolite in PEBA matrix enhanced permselectivity of the membranes such that high selectivity at a reasonable flux was obtained.

In a study carried out by Najafi et al. [123], hydrophobic graphene nanoplatelets were incorporated in PEBA polymer to prepare nanocomposite membranes for removal of isopropanol from aqueous solution by PV process. It was reported that the hydrophobic nature of graphene increased adsorption of isopropyl alcohol which led to an increase in separation factor and permeate flux.

### 5.3. Organic-Organic Separation by Target-Organophilic Nanocomposite Membranes

Among different applications of PV, separation of organic/organic mixtures is more challenging because the components in the feed mixture have similar physicochemical properties and effective separation of these mixtures could be very difficult [16,20]. In this regard, when one of the components contains polar groups while the other is entirely nonpolar, the thumb rule of hydrophilicity/hydrophobicity of the polymer can be applied to select the membrane material [6]. Otherwise, when both components have similar properties, small differences in polarity can be exploited to result in relatively good separation [6]. The development of new membrane materials, particularly in combination with inorganic nanoparticles, can widen this application to PV [16]. Up to the present time, few studies investigated PV membranes to separate organic/organic mixtures (as reported in Table 9). This could be caused by two reasons: Firstly, polymers with excellent chemical resistance to aggressive mixtures are scarce, and secondly, it is difficult to tune membranes for each distinct application [16,20].

Shen et al. [30] incorporated Ag^+^/MWCNTs into a CS membrane for separation of benzene/cyclohexane mixtures by PV. They found that with increasing MWCNTs-Ag^+^ content, the flux and selectivity of membranes increased. They argued that this could be due to stronger interaction of benzene molecules with Ag^+^ in the nanocomposite membrane. In another study, Peng et al. [34] prepared CS-wrapped MWCNT/PVA membranes and investigated their PV performance for benzene/cyclohexane separation. After incorporating nanotubes into the PVA matrix, both permeation flux and separation factor of membranes increased. They reported that nanocomposite membranes showed higher solubility selectivity in comparison with pure PVA membrane due to stronger affinity of CNT towards benzene than cyclohexane. In another study, Penkova et al. [29] studied PV transport properties of poly (phenylene isophthalamide)/CNT nanocomposite membrane in separation of methanol/ methyl tert-butyl ether (MTBE) mixture. As compared to pure membrane, it was found that the nanocomposite membrane is more selective towards methanol. However, by increasing CNT loading in the membrane, selectivity decreased due to the presence of bigger agglomerates of CNT in the polymer matrix.

Similarly, Tamaddondar et al. [83] studied separation of methanol/MTBE mixtures by PV process using self-assembled polyelectrolyte surfactant complex (PELSC) nanocomposite membranes containing nano-sized SiO_2_ particles. It was observed that permeability increased by increasing nanoparticle content in the membranes, while selectivity decreased due to possible nonselective voids at the PELSC/nanoparticle interface. In a study done by Garg et al. [32], PV separation performance of azeotropic toluene/methanol mixture using PDMS/clay nanocomposite membranes was investigated. Their results showed higher selectivity of nanocomposite membranes for toluene as compared to pure PDMS membrane. However, permeation flux of these membranes reduced since the polymer chains surrounding the clay were restricted by nano-sized clay such that the diffusion of small molecules through the nanocomposites was reduced. Wang et al. [88] studied PV performance of nano-silica/PDMS nanocomposite membrane for dimethyl carbonate (DMC) removal from DMC /methanol mixture. It was found that by filling membrane with inorganic particles, membrane separation performance improved in terms of higher separation factor while permeation flux decreased. They concluded that this result could be attributed to restricted plasticization of membrane.

Polotskaya et al. [98] investigated PV separation of methanol from binary organic mixtures (methanol/toluene and methanol/cyclohexane) using PI/PANI nanocomposite membrane. In both PV processes, nanocomposite membranes performed better in terms of higher selectivity, while permeation flux was lower in these membranes. The observed result was due to decreased sorption ability of membranes with increase in polymer–solvent interaction parameter values for PI–PANI.

In a recent study carried out by Ding et al. [126], carbon nitride nanosheets/PEBA membranes were prepared and used for PV separation of octane-thiophene mixture. In this work, Cu^+^ and Fe^2+^ ions were co-impregnated onto carbon nitride nanosheets and then the resultant modified nanosheets were incorporated into the PEBA matrix to facilitate thiophene transport. It was found that both permeation flux and enrichment factor were higher compared to that of pure PEBA membrane) because of reversible interaction between Cu^+^ and thiophene, which improved selectivity of membranes. Moreover, the presence of Fe^2+^ stabilizer could prevent disproportionation and oxidation of Cu^+^.

## 6. Conclusions and Future Directions

This review presents recent progress of research on PV nanocomposite membranes, focusing on the preparation methods, the effect of nanoparticles on membrane properties, and membrane performance in separation process. Three main methods have been used to prepare nanocomposite membranes for PV applications including solution casting, in situ polymerization, and sol-gel method. Among these methods, solution casting is the most common method to fabricate polymeric nanocomposite membranes because it can be used for all kinds of nanoparticles. Moreover, concentrations of polymer and nanoparticles can be controlled easily. However, this method has some disadvantage such as aggregation of nanoparticles in membrane structure as well as development of voids between polymer and nanoparticle phases. Some methods which have been used to modify membrane fabrication procedure to prepare membranes with higher separation efficiency were described.

The effects of incorporation of nanomaterials into polymers on the membrane structure and other properties of membranes such as free volume, crystallinity, swelling degree, and hydrophilicity/hydrophobicity were reviewed. Various changes have been reported in the literature in this regard. In general, nanocomposite membranes have shown many excellent promising properties for PV applications. A literature review was also conducted on the application of membranes in the PV process. Generally, the reports can be classified in three categories: separation of water from organic/water solutions, separation of organics from organic/water solutions, and organic-organic separation.

In terms of membrane manufacture, there are still issues that should be overcome in the future. The agglomeration of nanomaterials, which is detrimental to performance of the resultant membranes, should be addressed using cost-effective alternatives. Functionalization of nanomaterials has been introduced, but there is still a long way to go in this field. Moreover, PV separation of liquids in the industrial scale must be studied and compared with conventional alternatives. The environmental aspects of end-of-use PV membranes should also be considered. The application of biodegradable polymers and waste plastics as sustainable alternatives for preparation of PV membranes should be investigated in detail and clarified in the future.

## Figures and Tables

**Table 1:** PV nanocomposite membranes used in different applications.

**Dehydration of Aqueous-Organic Mixtures**
Poly (vinyl alcohol) (PVA)/iron oxide [76,81], PVA/SiO_2_ [45,73,75,86,100], PVA/CNTs [27,42,47,49,53,101], PVA/titanium dioxide (TiO_2_) [71], PVA/ZIF [62], PVA/nanoclay [94,102], PVA/fullerenol [51], Poly (vinyl amine)-PVA/CNTs [46], PVA-g-acrylonitrile/2-hydroxyethyl methacrylate (HEMA)/iron oxide [77], Crosslinked PVA/N-p-carboxy benzyl chitosan/Sulfonated SiO_2_ [87], PVA/graphene oxide-framework (GOF) [103], PVA/poly(HEMA) grafted SiO_2_ nanospheres [104], PVA-polyethersulfone (PES)/nano zeolite X/PVA [105], PVA-hydroxyethyl cellulose (HEC)/nanoclay [93] PVA/iron-dopamine (Fe-DA) nanoparticles [106], PVA-maleic acid (MA)/SiO_2_ [107], PVA/graphitic carbon nitride [108], PVA/MOF [109] Sodium alginate (SA)/CNTs [56], SA/TiO_2_ [36], SA/PTA [13], SA/amphiphilic carbonaceous material (ACM) [110], SA/zwitterionic GO [111], SA/POSS [112], SA/attapulgite nanorods [113], SA/bacterial cellulose [114] Chitosan (CS)/CNTs [48,54,55], CS/TiO_2_ [90], CS/iron ferroferric oxides [4], CS/SiO_2_ [78] CS/GO [115], CS/PVA-multiwalled carbon nanotubes (MWCNTs) [55], CS/prussian blue nanoparticles [116], CS/titanate nanotubes (TNTs) [44], CS/titanosilicate [21] PA/nanoclay [37], PA/zeolite [10] PI/SiO_2_ [7], PI/zeolite [7], PI/magnesium oxide (MgO) [3], PI/iron oxide [22], PI/ZIF [63], PI/zirconium dioxide (ZrO_2_) nanostars [117] Polysulfone (PSU)/nano-iron [28] Acrylonitrile-butyl acrylate copolymer/nanoclay [96] Polybenzimidazole (PBI)/ZIF [65,66] Polyphenylsulfone (PPSU)/SiO_2_ [72] Humic acid-like polymer (HAL)/GO [118] Polyelectrolyte complex (PEC)/GO-CNT nanofillers [119]
**Organics removal from aqueous-organic mixtures**
Poly (dimethylsiloxane) (PDMS)/silica (SiO_2_) [26,41,60,69,79,82,92,120], PDMS/silicalite [58,59,60,61], PDMS/zeolite imidazolate framework (ZIF) [67,68,69,121], PDMS/ polyphosphazene nanotube (PZSNTs) [99], PDMS-Polyamide (PA)/SiO_2_ [85], PDMS/activated carbon nanoparticles [122] Poly (ether block amide) (PEBA)/zeolite [12], PEBA/graphene [123], PEBA/polyhedral oligomeric silsesquioxane (POSS) [35,124] Natural rubber (NR)/SiO_2_ [84] PVDF/carbon black [52] Poly (methylphenylsiloxane) (PMPS)/ZIF [64] Butyl acrylate-styrene copolymer/nanoclay [95] Polymer of intrinsic microporosity (PIM)/graphene oxide (GO) derivatives [125] Poly (2,6-dimethyl-1,4-phenylene oxide) (PPO)/fullerene [50]
**Separation of organic-organic mixtures**
PVA/carbon nanotubes (CNTs) [34], PVA/MOF [70] PDMS/SiO_2_ [88,89], PDMS/nanoclay [32] CS/CNTs [30] Poly (phenylene isophtalamide)/CNT [29] Polyimide (PI)/polyaniline (PANI) [98] PEBA/carbon nitride nanosheets [126] Polyelectrolyte surfactant complex (PELSC)/SiO_2_ [83]

**Table 2:** PV nanocomposite membranes prepared using the solution blending method reported in the literature.

Polymer	Nanofiller	Casting Method	Blending Technique	Description	Ref.
PDMS	Silicalite	Dry	(a)	Drying in a vacuum oven at 120 °C	[59]
PEBA	Zeolite	Dry	(a)	Drying at room temperature initially and then heating in a vacuum oven at 70 °C	[12]
PI	Aerosil SiO_2_	Dry	(a)	After solvent evaporation, films were peeled at 170 °C to avoid film rapture and then they were placed between two stainless steel meshes on oven	[7]
	Zeolite		(a)		
PI	MgO	Dry	(a)	High-processing temperature was applied during membrane formation (drying at 170 °C in a vacuum oven under N_2_ flow and heating up to 200 °C, and then annealing at 250 °C)	[3]
PEBA	Graphene	Dry	(a)	Drying in an oven at 60 °C for 24 h	[123]
PDMS	SiO_2_	Dry	(b)	Drying at room temperature initially and then in a vacuum oven at 80 °C	[92]
PVA	SiO_2_	Dry	(b)	Drying at room temperature initially and then in a vacuum oven at 140 °C	[86]
PVA	ZIF	Dry	(b)	Drying in an oven at 40 °C	[62]
PDMS-PA	Fumed-SiO_2_	Dry	(b)	Drying at room temperature initially and then in a vacuum oven at 60 °C	[85]
PVA	Acid functionalized MWCNTs	Dry	(b)	Drying at 40 °C	[47]
PVA	Bentonite clay	Dry	(b)	Drying in a hot air oven at 40 °C	[94]
SA	PANI-TiO_2_	Dry	(b)	Drying at room temperature, then immersion in a bath for crosslinking, washing with distilled water and again drying at room temperature	[36]
CS	MWCNTs	Dry	(b)	Drying at room temperature	[54]
PI	Iron oxide	Dry	(b)	Drying at 50 °C and then at 70 °C in a vacuum oven	[22]
SA/poly (vinyl pyrrolidone) blend polymers	PTA nanoparticles	Dry	(b)	Drying at room temperature	[33]
PDMS	Activated carbon	Dry	(b)	Drying under vacuum conditions first at room temperature for 30 min and then at 90 °C for 3 h	[122]
PVA	Poly (HEMA)-grafted SiO_2_ nanospheres	Dry	(b)	Drying at 70 °C in an oven for 2 h	[104]
PVA	SiO_2_	Dry	(b)	Dry slowly in a dust-free atmosphere at ambient temperature	[75]
PEBA	POSS	Dry	(b)	Drying at room temperature and then in an oven at 120 °C for 3 h	[124]
PVA	MOF	Dry	(b)	Drying in an oven at 40 °C overnight	[109]
CS	Cyano-bridged coordination polymer nanoparticles	Dry	(b)	Drying in an oven at 45 °C overnight	[116]
PDMS	Modified fumed SiO_2_ particles	Dry	(c)	Drying at room temperature initially and then in an oven at 80 °C	[79]
PVA	CNTs	Dry	(c)	Drying at room temperature initially and then annealing	[42]
PVA	Poly (sodium 4-styrenesulfonate) (PSS)-wrapped MWCNTs	Dry	(c)	Drying at room temperature and then annealing at 90 °C	[27]
PVA	CNTs	Dry	(c)	Drying at room temperature and In situ crosslinking	[53]
PVA	Pure MWCNTs, functionalized MWCNTs, TiO_2_-MWCNTs	Dry	(c)	Drying in an oven at 80 °C initially and then heating at 150 °C	[49]
PDMS	ZIF	Dry	(c)	Drying at room temperature initially and then heating at 80 °C for further crosslinking	[68]
PVA	PANI-TiO_2_	Dry	(c)	Drying at room temperature, then cross-linking in a bath, soaking in distilled water after cross-linking	[71]
PDMS	PZSNTs	Dry	(c)	Drying at room temperature, crosslinking at room temperature, and then under vacuum at 50 °C	[99]
PPO	Fullerene (C_60_)	Dry	(c)	Drying in a vacuum oven at 40 °C	
PEBA	POSS nanoparticles	Dry	(c)	Drying at room temperature initially and then heating in a vacuum oven at 50 °C	[35]
SA	PTA in unmodified and modified form (by ammonium carbonate)	Dry	(c)	Drying at room temperature, immersion in a cross-linking solution bath, washing with deionized water and again drying in hot air oven at 40 °C	[13]
CS	Titanosilicate	Dry	(c)	Drying at room temperature initially and then heating in a vacuum oven at 120 °C	[21]
CS	Poly (aspartic acid)-modified TNTs	Dry	(c)	Drying at room temperature	[44]
HAL	GO	Dry	(c)	Drying at room temperature for three days and then in a vent oven at 45 °C for 24 h	[118]
PEBA	Cu^+^ and Fe^2+^ ions co-impregnated carbon nitride	Dry	(c)	Drying overnight at 30 °C and then at 60 °C for 24 h	[126]
cardo polyetherketone (PEK-c)	ZIF nanocrystals	Dry	(c)	Drying at 60 °C for 36 h and further drying in a vacuum oven at 120 °C for 12 h	[110]
PVA	Fe-DA nanoparticles	Dry	(c)	Drying at room temperature for 24 h and then at 120 °C for 1 h in a vacuum oven	[106]
PVA	SiO_2_ nanospheres	Dry	(c)	Drying at room temperature for 24 h and then in an oven at 40 °C for 18 h.	[100]
PVA	Graphitic carbon nitride	Dry	(c)	-	[108]
PI	ZrO_2_ nanostars	Dry	(c)	Drying for 12 h at 80 °C	[117]
PVA	Acid-treated MWCNTs	Dry	(c)	Drying at room temperature for 48–72 h and heating in a vacuum oven at 150 °C for 1 h	[101]
PSU	Bentonite clay	Dry	_-*	Drying in an oven at 80 °C initially and then in a vacuum oven	[28]
CS	Ag^+^/CNTs	Dry	-	Drying at room temperature initially and then at 40 °C	[30]
PVA	CS-wrapped CNT	Dry	-	Drying at room temperature	[34]
PVA	Cellulose nanocrystals	Dry	-	Drying at room temperature	[31]
PVA	Low-hydroxylated fullerenol (C_60_(OH)_12_)	Dry	-	Physically cross-linked by heating at 140 °C and chemically cross-linked by heating at 110 °C	[51]
PDMS	MCM-ZIF hybrid particles	Dry	-	Drying at room temperature initially and then in a vacuum oven at 90 °C for complete cross-linking	[67]
PDMS	Organophilic SiO_2_	Dry	-	Drying at room temperature	[82]
NR	Styrene butadiene copolymer (SBR)-SiO_2_ nanoparticle	Dry	-	Heating in an oven at 40 °C	[84]
PELSC	SiO_2_	Dry	-	Drying at 50 °C	[83]
PDMS	Functionalized fumed SiO_2_	Dry	-	Drying at room temperature initially and then in a vacuum oven at 80 °C	[41]
PEC	SiO_2_	Dry	-	Drying at 60 °C	[91]
PDMS	SiO_2_	Dry	-	Drying at room temperature initially and then at 120 °C for complete cross-linking	[88]
PI	PANI	Dry	-	Drying in vacuum at 50 °C	[98]
PIM	Functionalized GO	Dry	-	Drying at room temperature in a drying cabinet with continuous flow of N_2_	[125]
PSU	Iron	Dry	-	The cast film was kept in an oven for 2 h at 80 °C and then in a vacuum oven for 24 h	[28]
PVA-HEC	Hydrophilic bentonite	Dry	-	Drying at room temperature and then at 110 °C in a hot air oven for crosslinking reaction	[93]
PDMS	SiO_2_	Dry	-	Drying slowly under ambient conditions for 48 h and then in an oven at 85 °C for 2 h	[26]
PDMS	Two commercial clays (functionalized with polar and nonpolar surfactants)	Dry	-	Heating at 150 °C and then annealing the films at 200 °C In situ crosslinking	[32]
PVDF	Carbon black	Dry-wet	(a)	Drying at room temperature initially, immersion in three precipitation baths, and again drying at room temperature	[52]
PDMS	SiO_2_/Silicalite-1 particles	Dry-wet	(b)	Drying at room temperature initially and then in an oven at 70 °C for complete cross-linking, immersion in water bath and again drying in the oven at 70 °C	[60]
SA	Mesoporous molecular sieve (MCM)	Dry-wet	(c)	Immersion in a crosslinking bath, washing with deionized water and then drying in an oven at 40 °C	[57]
PBI	ZIF	Dry-wet	(c)	Drying in a vacuum oven at 75 °C and cooling down, then further drying in a vacuum oven at 200 °C, and solvent-exchanging with methanol and again drying in a vacuum oven at 200 °C	[65]
PBI	ZIF	Dry-wet	(c)	Two steps heating in vacuum oven and cooling down naturally, then solvent-exchange with methanol, and heating in vacuum oven once more	[66]
PI	ZIF	Dry-wet	(c)	Heating in an oven at 60 °C and then solvent-exchange with methanol and again drying in a vacuum oven at 60 °C	[63]
CS	PVA-MWCNT	Dry-wet	-	Drying at room temperature initially and then immersion in an alkaline solution followed by washing with deionized water and again drying at room temperature	[55]
PPSU	SiO_2_	Wet	(a)	Immersion in a non-solvent (water)	[72]
PVA	Polydopamine-coated MOF	Wet	-	Drying at room temperature for 24 h and then drying at 80 °C under vacuum for 36 h	[130]
PDMS	ZIF	Wet	-	Drying at room temperature for 48 h and then heating at 100 °C for 15 h under vacuum	[121]
SA	MWCNT decorated by Fe_3_O_4_ nanoparticles	Wet	Spin-coating	Spin-coating of a blend of SA solution containing Fe_3_O_4_-CNT nanofillers onto PAN membranes	[56]
Polyvinylamine-PVA	CNTs	Wet	Solution coating technique	Heating in an oven at 55 °C after solution coating	[46]
SA	Attapulgite nanorods	Wet	Blending and spin coating methods	-	[113]
PDMS	SiO_2_	Wet	Sonication-enhanced in situ assembly	Drying at the ambient temperature initially and then in a convection oven at 80 °C	[69]
	ZIF				
PDMS	Silicalite	Wet	Dip-coating process	Heating the casting solution to 65 °C with continuous mixing for 180 min and then cooling to 25 °C for dip-coating	[61]

* Not mentioned.

**Table 3:** PV nanocomposite membranes prepared using polymerization method reported in the literature.

Polymer	Nanofiller	Polymerization Method	Description	Ref.
PVA	PANI	In situ polymerization	Polymerization of aniline in PVA matrix in acidic media	[97]
PA	Zeolite	Interfacial polymerization	- *	[10]
Poly (methyl methacrylate (MMA)-co-acrylamide (AM))	AgCl nanoparticles	In situ microemulsion polymerization	-	[132]
Poly (AN-co-BA)	Sodium montmorilonite	Emulsion polymerization	Drying at 60 °C and then annealing at 80 °C	[96]
SBR	Organophilic clay	Emulsion polymerization	Incorporating the clay in the polymer during its synthesis from the butyl acrylate and styrene monomer by emulsion polymerization in water	[95]
PA	SDS-clay	Direct polycondensation	Direct polycondensation in the presence of organo-modified montmorillonite (organo-clay) in *N*-methyl-2-pyrrolidinone (NMP)	[37]

* Not mentioned.

**Table 4:** PV nanocomposite membranes prepared using the sol-gel method reported in the literature.

Polymer	Nanofiller	Description	Ref.
Cross-linked PVA-(N-o-sulphonic acid benzyl chitosan) (NSBC)	Sulfonated SiO_2_	Using the sol-gel method followed by oxidation of the thiol group into the sulfonic acid group	[87]
PVA	SiO_2_	Drying at room temperature initially and then at higher temperatures (80 °C and 150 °C respectively)	[80]
N-p-carboxy benzyl chitosan (NCBC)	Functionalized SiO_2_ Sodium 2-formylbenzenesulfonatepolysiloxane (SBAPTS)	Sol-gel followed by cross-linking under different reaction conditions	[78]
PVA	Mercaptopropyltrimethoxysilane (MPTMS)	Drying at room temperature, annealing at 80 °C, then heating at 150 °C and oxidizing by hydrogen peroxide in an acetone solution, then washing the resulting membranes with distilled water	[45]
CS	TiO_2_	Drying at room temperature and then annealing at 120 °C	[90]
PVA	Iron oxide	The reaction of oxide formation taking place inside the polymer matrix itself	[76]
PVA-g-acrylonitrile/HEMA	Iron oxide	Adding Fe^2+^ and Fe^3+^ solutions into PVA-g-acrylonitrile/HEMA polymer solution, stirring and then casting onto rimmed round glass dishes, drying at 80 °C, immersion in a bath containing KOH solution for 24 h for in situ synthesis of Fe_3_O_4_ in the membrane matrix, washing with distilled water repeatedly until neutrality	[77]
PVA	Iron oxide	Filtering an aqueous solution containing FeSO_4_ and FeCl_3_to remove iron hydroxide and adding the solution to PVA aqueous solution and then stirring at ambient temperature. casting the resultant solution, drying at ambient temperature followed by immersion in a bath containing KOH solution for 24 h. Finally washing repeatedly by immersion in double distilled water until neutrality and drying at the ambient temperature	[81]
PEC	GO-CNT	- *	[119]
PVA-MA	SiO_2_	Drying in air and then heating in an oven at 140 °C for 2 h	[107]

* Not mentioned.

**Table 5:** PV nanocomposite membranes reported in the literature prepared using methods other than blending, polymerization, and sol-gel.

Polymer	Nanofiller	Preparation Method	Description	Ref.
PDMS	Silicalite	packing-filling	Silicalite nanocrystals were deposited onto a porous alumina capillary support using dip-coating technique (packing); then, the interspaces among the nano-crystals were filled with PDMS phase (filling)	[58]
PVA	MOF-based CU_3_(BTC)_2_ (BTC = benzene-1,3,5-tricarboxylate)	pressure-driven assembly	The Cu_3_(BTC)_2_/PVA membrane solution was assembled onto amino ceramics substrate	[70]
Poly (phenylene isophtalamide)	CNT	solid phase interaction	Mixing of powders of PA and nanotubes In porcelain mortar and after the solid-phase interaction, Dissolving the composites in solvent, Drying at room temperature initially and then heating in a vacuum oven	[29]
Silicone rubber	ZIF	plugging-filling method	Plugging the holes in the top layer of the hierarchically ordered stainless steel mesh (HOSSM) with ZIF nanoparticles, and then filling the spaces between the nanoparticles and mesh wires with PMPS silicone rubber	[64]
CS	Poly (3-Hydroxybutyrate) (PHB)-functionalized MWCNTs	filtration method	The bulk alignment of PHB-MWCNT in the CS matrix by filtering the PHB-MWCNT/chloroform solution through a polytetrafluoroethylene (PTFE) membrane and then coating the membrane filter with the CS solution and drying at room temperature	[48]
SA	Zwitterionic GO	spin-coating method	-	[111]
SA	POSS	spin-coating method	-	[112]
Poly (diallyl dimethyl ammonium chloride) (PDDA)/PSS and poly (ethyleneimine)/polyacrylic acid	ZrO_2_ and Al2O3 nanoparticles	layer-by-layer assembly method	Incorporation of single-component nanoparticles into both the polycation and polyanion layers, and assembly of nanohybrid multilayer on both flat sheets and hollow fiber porous substrates	[74]

**Table 6:** The main changes in the morphology, swelling degree, and water contact angle of the nanocomposite membranes.

Polymer	Nanoparticle	Main Effects of Nanoparticles on Membrane Morphology	Changes in the Swelling Degree of Nanocomposite Membranes	Changes in Contact Angle of Nanocomposite Membranes	Ref.
PVA	Silica	The heterogeneity between the agglomerated particles and the polymer in the membrane structure Reduction in crystallinity of the membrane and development of amorphous regions in the polymer	**Swelling degree:** From ~27% for pure membrane to ~40%	— *	[86]
			Description: Nanoparticles enhanced the amorphous regions in the polymer matrix Increase in the surface hydrophilicity of hybrid membranes due to the presence of silica nanoparticles in the polymer matrix		
		Destroying the formation of crystalline regions	—	Water contact angle: From 58.58° for pure membrane to 46.91°	[45]
		Lower degree of crystallinity in the nanocomposite membrane More compact polymer chains and cross-linked networks are formed in the membrane structure	—	Water contact angle: From 66.8° for pure membrane to 64.8°	[80]
				Description: Increase in the hydrophilicity of membrane due to the higher number of hydroxyl groups attached to the polymer-silica matrix	
PVA	sulfonated silica	Reduction in void porosity in the membrane matrix	Less swelling ratio in comparison with pristine membrane Description: Decrease in the mobility of polymer chains in water	Lower contact angle for water (68–70°), in comparison with ethanol (85–87°)	[78]
PVA	Iron oxide	More compact structure and increase in the amorphous characteristics of the membranes with iron contents	~10% for nanocomposite membrane	—	[76]
		Formation of more amorphous regions Reduction in the relaxation of polymer chains	Swelling degree: For water/ isopropanol mixture: ~19% For water/ 1,4-dioxane mixture: ~15% For water/ THF mixture: ~25%	—	[81]
			Description: Each iron ion formed a network structure with hydroxyl groups of PVA so that the membrane swelling reduced		
PVA	MWCNT	Formation of a network-like structure	—	Water contact angle: from 72° for pure membrane to 63°	[47]
				Description: Reduction in the hydrophobic nature of the cross-linked PVA membranes	
PVA	CS -wrapped CNTs	Increase in the free volume Providing internal nanochannels for permeation from the nanoscale opening of CNTs	—	—	[34]
PVA	PSS wrapped MWCNTs	More rigid polymer matrix	Swelling degree: From ~35% for pure membrane to ~25%	—	[27]
			Description: Decrease in the polymer chain mobility as a result of the good interaction between PVA and MWNTs		
PVA	PANI	Increase in the free volume space	Equilibrium swelling (g): from 0.114 for pure membrane to 0.084	—	[97]
			Description: Increase in void spaces in the nanocomposite membranes and the water-adsorptive nature of the PANI particles		
PVA	Cellulose	Proficient adhesion between the two constituents and no extra sieve-in-a-cage phenomena formed in the film	—	—	[31]
PVA	Nanoclay	Formation of a well exfoliated structure in the nanocomposite membranes Reduction in the rigidity of the polymeric chain Increase in the amorphous region of the membranes An increase in their tensile strength as compared to pristine PVA membranes	Swelling degree: 102 ± 2%	Water contact angle: From 63.00 ± 2 for pure membrane to 58.66 ± 2	[94]
CS	TiO_2_	Increase in the flexibility of polymer chains	Swelling degree: From 6% for pure membrane to ~9.5%	From 42° for pure membrane to 56°	[90]
			Description: Increase in the interaction between water molecules and nanoparticles so that the adsorption of hybrid membranes increased for water molecules		
CS	Iron oxide	Formation of the three-dimensional network structure of iron with hydroxyl and amine groups of chitosan	Swelling degree: From ~67% for pure membrane to ~10%	—	[4]
			Description: Change in the hydrophilic–hydrophobic balance of the polymer matrix resulting a reduction in membrane swelling		
CS	PHB-functionalized MWCNTs	Enhancement in the tensile strength of the chitosan matrix Decrease in the flexibility of membranes The bulk alignment of the PHB-MWCNT	For the feed mixture: from ~0.25 for pure membrane to ~0.15 (g/g membrane) For water: from ~1.9 to ~0.9 (g/g membrane)	—	[48]
			Description: Restricted sorption capacity due to the reduction in the flexibility and increase in the hardness of the nanocomposite membrane		
CS	MWNTs–Ag^+^	More loosely packed chitosan chains Decrease in the crystallinity of membrane Change in the aggregation structure of chitosan Increase in the free volume of the hybrid membranes	From ~4% for pure membrane to ~9.2%	—	[30]
CS/PVDF	PVA–MWCNT	Decrease in the flexibility of polymer chains and reduction in the free volume of the polymer matrix	Swelling degree: From ~0.05 for pure membrane to ~0.025 (g/g membrane)	—	[55]
CS			Description: Decrease in the sorption capacity of the nanocomposite membrane due to the ability of PVA–MWCNT to restrain the mobility of the polymer chains through the interaction between the functional groups of PVA–MWCNT and the polymer molecules		
SA	Bacterial cellulose	Denser structure Enhanced hydrophilicity	44.2%	53	[114]
SA	Fe_3_O_4_@CNT	Decrease in the mobility of polymer chains and larger interaction between the polymer and inorganic particles	Swelling degree: From ~5.5% for pure membrane to ~4.5%	From ~23° for pure membrane to ~25°	[110]
SA	PTA	A rougher surface than the pristine NaAlg The structural strength improved Plasticization of the polymer was reduced The void spaces of the polymer were filled up	Swelling degree: From ~20% for pure membrane to ~10% in water–isopropanol mixture From ~15% for pure membrane to ~5% in water–ethanol mixture	—	[13]
SA			Description: Increased swelling in water–ethanol due to interactions of free terminal hydrophilic –OH groups of ethanol with NaAlg Reduction of membrane swelling as the matrix becomes more rigid		
SA	MCM	—	Swelling degree: From ~2% for pure membrane to ~2.5%	—	[57]
			Description: The filler particles will help the hybrid matrix to absorb more of water molecules		
silicone rubber	ZIF	Homogeneity in the membrane structure and no interfacial voids between the mesh wires and the ZIF-8 nanoparticles in the PMPS phase	—	—	[64]
PDMS	Silicalite	Void formation at the particle/polymer interface	—	—	[61]
PDMS	SiO_2_	Hierarchical roughness surface	—	Water static contact angle: 152 ± 0.6°Ethanol contact angle: Less than 8°	[92]
PDMS				Description:Increase in the wettability of membrane due to the enhanced surface roughness	
PDMS	PZSNTs	Well dispersion of nanotubes in PDMS	Swelling degree: For ethanol: from 5.6% for pure membrane to 24% For water: from 0.6% for pure membrane to 2% For the feed mixture: from 1.8% for pure membrane to 7.5%	—	[99]
PDMS	Silica	Dense structure with no appreciable voids at the interface of the PDMS and nanosilica particles	Swelling degree: From 2% for pure membrane to 4%	Water contact angle: From 109.0° for pure membrane to 116.5°	[82]
			Description: Increased accessible free volume in the matrix facilitates the sorption of water and ethanol molecules		
PDMS	Clay	The clays were oriented in a particular fashion rather than randomly A non-porous, tight and dense structure with no pinhole, connected pores or crack	—	—	[32]
PDMS	Silicalite	Dense structure with uniformly distributed particles in the membranes	—	—	[63]
PDMS-PA	Fumed silica	Deposition of the fumed silica particles near the interface of the PDMS skin layer The disruption of PDMS chain packing by the introduced fumed silica Increase in the free volume of PDMS Production of more defects in the skin layer	—	—	[85]
PDMS	Silica	Dense structure with no macroscopic voids Increase in the surface roughness of nanocomposite membranes Silica made the polymer chains more rigid and reduced free volume of polymer Higher polymer chains packing	Swelling degree: From 24% for pure membrane to 7%	—	[79]
			Description: Increase in the membrane surface roughness influences the absorption of penetrants		
PA	SDS–clay	The SDS–clay was intercalated and exfoliated into the polyamide matrix	Swelling degree: From 28% to 19%	—	[37]
			Description: Decrease in degree of swelling due to the resistance of the molecular diffusion and tortuosity of the diffusion pathway		
PA	Zeolite	Increased roughness of the membrane by adding nano NaX zeolite	—	From 65 ± 2.3° for pure membrane to 32±1.3°	[10]
NR	SBR–SiO_2_	Improvement in the tensile strength and modulus of the nanocomposite membrane Reduction of the tight packing of polymer chains due to the highly dispersed silica nanoparticle in the membrane	—	From 110.1 ± 0.3° for pure membrane to 77.4 ± 2.6°	[84]
				Description: An enhanced effect on the hydrophilic surface of the membrane due to the reactive hydroxyl groups of the SBR–SiO2 nanoparticle exhibited	
PELSC	SiO_2_	An increase in free volume as well as the possible defects at the interface of polymer and silica clusters	—	Description: No image could be taken due to the great hydrophilicity	[83]
PEBA	Zeolite	The crystallinity of membrane increased Homogenous dispersion of zeolite in a systematic and uniform pattern throughout the surface with absence of any voids in the polymer film	The equilibrium swelling ratio: ~6	—	[12]
PBI	ZIF	Increase in the free volume of membrane	Sorption (g solvent/g membrane): In water: from 0.078 for pure membrane to 0.058 In methanol: from 0.155 for pure membrane to 0.206 In ethanol: from 0.172 for pure membrane to 0.198 In n-butanol: from 0.012 for pure membrane to 0.109	—	[65]
			Description: The hydrophobic nature and rigid structure of ZIF particles led to a decrease in the degrees of membrane swelling in water Increase in the n-butanol-induced swelling due to a greater free volume in the PBI/ZIF membrane		
PI	Zeolite	No voids (at least connected) around the nanoparticles inside the polymer matrix Uniform distribution of nanoparticles without serious agglomeration within polymer matrix Disturbance in the tight alignment of polymer and formation of the hydrophilic channels More plasticizable structure	—	—	[7]
PI	Silica aerosol				
PI	MgO	A disruption in the polymer chain packing due to the presense of nanosize crystals on the MgO surface A densely packed structure at both top side and bottom side Rigid polymer chains surrounding the particle Rougher and more nodule type surface due to the fast evaporation of solvent	Swelling degree: From 0.02 for pure membrane to 0.027 g solution/g membrane. Description: The hydrophilic channels were seriously swollen (plasticized) by the water.	—	[3]
PI	PANI	—	The equilibrium swelling: In methanol: from 12.5% for pure membrane to 10.6%. In toluene: from 7.7% for pure membrane to 6.9%. In cyclohexane: no swelling	Water contact angle: From 87.4° for pure membrane to 90.5°	[98]
			Description: The presence of rigid PANI particles in the polymer matrix decreased the equilibrium swelling or sorption for both methanol and toluene		
PVA and HEC	Nano bentonite	The loss in crystallinity of the blend and filled membranes Higher tensile strength but lower elongation at fracture than unfilled blend membranes Increase in the stiffness of membranes in presence of inorganic fillers	Sorption in water: From 15% for pure membrane to 25%	—	[93]
Poly (styrene-co-butyl acrylate)	Nanoclay	Much higher tensile strength than the unfilled membranes Elongation properties of the membrane decrease Uniform mixing without any significant stress concentration at the filler–polymer interfaces	—	—	[95]
PEBA	POSS	Dense and homogenous structure with no bulky agglomeration Disruption of inherent organization of polymer chains The polymer strength reduced and the accessible free volume in the polymer matrix enhanced	Description: The membrane swelling and the degree of sorption decreased due to the POSS–polymer chain interactions in the solvent solution		[35]
PVA	Bentonite clay	Formation of a well exfoliated structure in the cross-linked PVA nanocomposite membranes Higher thermal stability Reduction in the rigidity of the polymeric chain is with the addition of nanoclay Increase in the tensile strength as compared to pristine PVA membranes Increase in the amorphous region of the membranes with the addition of nanoclay	Description: With an increase in the nanoclay content, the polymer chains become less mobile due to the reduced swelling	Water contact angle: From 63.0° for pure membrane to 58.6°	[94]
PPSU	SiO_2_	A pore size reduction while closing some of the other smaller pores that are visible in PPSU membranes Decrease in the porosity of the membranes, simultaneously obtaining an improved hydrophilicity Formation of denser membranes membranes with less number of pores and smaller pore sizes More hydrophilic membrane with higher surface roughness		Water contact angle: From 65 for pure membrane to 48° particles modify the structure of the membrane and the porosity of the top layer, which closes any pores formed and thus increases the contact angle of the modified membrane	[72]
PVDF	Carbon black	Carbon black particles filled the free spaces of sponge like structure of the PVDF membrane and formed a denser structure The crystallinity of the filled membrane increased	Swelling in feed mixture: From 18.47% for pure membrane to 0.91%	Water contact angle: From 84.7° for pure membrane to 90.7°	[52]
			Description: The lower swelling degree in the nanocomposite membrane was attributed to denser structure of the filled membranes	Description: Hydrophobic nature of carbon black particles increased the hydrophobicity of membrane	

* Not mentioned.

**Table 7:** PV performance of hydrophilic nanocomposite membranes.

Polymer	Nanoparticle	Feed Solution and Composition (wt. Ratio)	Operating Temperature (°C)	Downstream Pressure (Pa)	Separation Factor (α)	Total Flux (kg/m^2^.h)	PSI	Ref.
PVA	MWCNTs	water/ethanol (4.37/95.63)	room temperature	266.64	160	~0.075	~11.5	[47]
PVA	Iron oxide	water/acetonitrile (10.7/89.3)	35	100–1500	711.12	0.0129	9.1747	[76]
PVA	MWNTs-PSS	water/IPA (10/90)	30	666.61	882	0.168	148.1	[27]
PVA	SiO_2_	water/ethylene glycol (20/80)	70	799.93	311	0.067	20.77	[45]
PVA	PANI	water/IPA (10/90)	30	1333.22	564.2	0.069	38.8608	[97]
PVA	Cellulose	water/ethanol (20/80)	80	500	163	~0.390	~63.57	[31]
PVA	Iron oxide	water/IPA (10/90)	30	6.67	470	0.079	37.051	[81]
		water/1,4 dioxane (10/90)			144	0.084	12.012	
		water/tetrahydrofuran (THF) (5/95)			519	0.095	49.21	
PVA	Aminopropyltriethoxysilane (APTEOS)/tetraethylorthosilicate (TEOS)-	water/ethanol (25/75)	50	1599.87	270	0.577	155.21	[80]
PVA	MOF	ethanol/water (92.5/7.5)	40	1915.84	~12	1.477	-	[109]
PVA	CNTs	water/IPA (10/90)	30	100	1794	0.079	141.32	[42]
PVA	GOF	water/acetic acid	30	-	131	0.289	37.859	[103]
PVA	Fe-DA	water/ethanol (10/90)	30	80,000	2980	0.995	2965.1	[106]
PVA	Clay	water/IPA (12.5/87.5)	room temperature	266.64	53	0.104	5.408	[94]
PVA	CNT	water/IPA (10/90)	30	100	1794.4	0.08	143.47	[53]
PVA	CNT	water/ethanol (10/90)	30	133.32	78	0.471	36.267	[49]
	Modified CNT				662	0.395	261.095	
	TiO_2_-CNT				805	0.388	311.952	
PVA	ZIF	water/IPA (10/90)	30	666.61	132	0.868	113.708	[62]
PVA	Fullerenol	n-butanol/water (90/10)	25	13.33	~250	~0.55	-	[51]
PVA	SiO_2_	water/caprolactam (30/70)	50	133.32	148	3.8	558.6	[75]
PVA	Graphitic carbon nitride	ethanol/water(90/10)	75	200	30.7	6.332	188.0604	[108]
PVA	Fe-DA	ethanol/water (90/10)	30	80,000	2980	0.995	2965.1	[106]
PVA	Polydopamine-coated MOF	ethylene glycol/water (90/10)	70	700	2864	0.540	1546.02	[130]
PVA/PES	nano zeolite X	ethanol/water (90/10)	30	133.32	178	0.374	66.572	[105]
PVA-g-acrylonitrile/HEMA	Fe_3_O_4_	water/acetone (20/80)	40	70	120	0.2	23.8	[77]
Polyvinylamine-PVA	CNT	water/ethylene glycol (1/99)	70	133.32	1156	0.146	168.63	[46]
double-network PVA	SiO_2_ nanospheres	water/ethanol (10/90)	30	100	17,800	0.006	106.8	[100]
HEC-PVA	Bentonite	water/THF (5.5/94.5)	30	133.32	195	0.112	21.728	[93]
CS	Titanosilicate	water/ethanol (15/85)	50	200	~30	~0.55	-	[21]
CS	TiO_2_	water/ethanol (10/90)	80	500	196	0.340	66.3	[90]
CS	MWNT	water/IPA (12.5/87.5)	30	66.66	296.2	0.50	147.6	[54]
CS	Cyano-bridged coordination polymer nanoparticles	ethanol/water (90/10)	25	-	1472	0.614	903.808	[116]
TNTs -embedded CS/PAN	TNTs	water/IPA (10/90)	80	300	6237	1.498	9341.528	[44]
CS /PVDF	PVA-MWCNT	water/acetone (5/95)	30	666.61	1450.5	0.089	129.0055	[55]
CS					1505.9	0.008	12.0392	
PHB/ CS	functionalised MWCNT	water/1,4 dioxane (5/95)	30–60	666.61–3333.06	623.11	0.017	10.5759	[48]
SA	Bacterial cellulose	water/ethanol (5/95)	40	1333.22	429.9	0.0333	14.1537	[114]
				266.64	612.1	0.109	66.6099	
SA	PANI-TiO_2_	water/1,4-dioxane (10/90)	30	33.33	12,848	0.034	436.798	[36]
SA	Attapulgite nanorods	ethanol/water (90/10)	76	100	2030	1.356	2750	[113]
SA	POSS	ethanol/water (90/10)	70	300	1077	2.500	2692.5	[112]
SA	zwitterionic GO	ethanol/water (90/10)	70	300	1370	2.140	2929.66	[111]
SA	MCM	water/IPA (10/90)	30	1333.22	29,991	0.11	3299.8899	[97]
SA/poly(vinylpyrrolidone)	PTA	water/ethanol (4/96)	27	500	7128	0.041	292.207	[33]
PA	Clay	water/ethanol (10/90)	25	-	~26	~0.285	-	[37]
	nano NaX zeolite	water/ethanol (10/90)	25	133.32	29.9	4.500	130.05	[10]
		water/isobutanol (10/90)			~325	~2.250	~729	
PI	SiO_2_ aerosil	water/IPA (10/90)	30	799.93	80	0.121	9.559	[7]
PI of matrimid 5218	zeolite				220	0.125	27.375	
PI of P84 s backbone	zeolite				890	0.159	141.351	
PI	ZIF	water/IPA (85/15)	60	100	5668	0.109	617.703	[63]
PI	ZrO_2_ nanostars	butanol/water (90/10)	40	10	109.3	0.140	15.302	[117]
PI	Iron (III) acetylacetonate (FeAc)	water/IPA (15/85)	60	100	4298	0.120	515.64	[22]
Polyacrylonitrile (PAN)	SA-Fe_3_O_4_-CNT	water/ethanol (10/90)	77	100	1870	2.211	4134.228	[56]
PAN	SA-CNT				551	1.832	1007.6	
PEC	GO-CNT	water/IPA (90/10)	40	-	~2750	~1.5	4092	[119]
PEC	SiO_2_	water/IPA (10/90)	70	-	2186	2.1	4588.5	[91]
PDDA/PSS	ZrO_2_	water/acetone (5/95)	50	100	361	1.240	446.4	[74]
NR/SBR-	SiO_2_	Water/ethanol (5/95 vol.%)		-	2976	0.425	1264.375	[84]
Iron	PSU	water/ethanol (10/90)	25	666.61–1066.58	~5000	~0.180	-	[28]
SiO_2_	PPSU	water/acetic acid (30/70)	70	-	3.3	2.34	5.382	[72]
PBI	ZIF	water/ethanol (15/85)	60	-	10	0.992	8.928	[66]
		water/IPA (15/85)			1686	0.103	173.555	
		water/butanol (15/85)			3417	0.081	276.696	

**Table 8:** PV performance of hydrophobic nanocomposite membranes.

Polymer	Nanoparticle	Feed Solution and Composition (wt. Ratio)	Operating Temperature (°C)	Downstream Pressure (Pa)	Separation Factor (α)	Total Flux (kg/m^2^.h)	PSI	Ref.
PDMS	Silicalite	ethanol/water (4/96)	25	200	16.5	~0.150	-	[61]
PDMS	SiO_2_	ethanol/water (4.8/95.2)	60	400–440	~6.5	~1	-	[92]
PDMS	PZSNT	ethanol/water (10/90)	40	1333.22	10	0.476	4.284	[99]
PDMS	ONS	ethanol/water (5/95)	60	100000	30.1	0.114	3.420	[82]
PDMS	Silicalite	acetic acid/water (20/80)	45	14.66–18.66	~2.4	~0.18	-	[59]
PDMS	SiO_2_	ethanol/water (5/95)	60	~200	12.5	0.807	9.2805	[69]
		n-butanol/water			9.2	1.693	13.8826	
PDMS	ZIF	ethanol/water (5/95)			9.9	1.229	10.9381	
		n-butanol/water			30.0	1.743	50.547	
PDMS	SiO_2_	ethanol//water (4/96)	50	100	26	0.329	8.225	[26]
		IPA/water (4/96)			31.7	0.405	12.4335	
PDMS	ZIF	butanol/water (1/99)	65	-	66	1.689	109.785	[68]
PDMS	Octyl-functionalized SiO_2_ (Si-DMOS)	1-butanol (1.5% wt./vol.)	60	3.07	3.4	~110	-	[41]
	Phenyl-functionalized SiO_2_(Si-DMPS)							
PDMS	SiO_2_	150 ppm toluene solution	23	100	3250	0.0000102	0.0331	[60]
PDMS	Silicalite	isobutanol/water (3/97)	80	-	25.0	11.2	268.8	[58]
PDMS	MCM/ZIF	ethanol/water (5/95)	60	-	7.6	1.029	6.7914	[67]
PDMS	MCM-ZIF	ethanol/water (5/95)	70		10.4	2.204	20.7176	
		n-butanol/water (3/97)	60		45	2.052	90.288	
PDMS	SiO_2_	ethanol/water (5/95)	40	10,000–30,000	19	0.200	3.6	[79]
PDMS	Activated carbon	butanol/water (0.5/95.5)	57	399.97	~23.5	~0.029	1.0762	[122]
PEBA	POSS (AL0136)	ethanol/water (5/95)	25	-	4.6	0.1835	0.6606	[35]
	POSS (SO1440)				4.1	0.1258	0.3899	
	Graphene	IPA/water (4/96)	50	2666.45	10.04	0.8425	7.619	[123]
	POSS	n-butanol/water (2/98)	40	100	27.2	1.33	36.176	[124]
SBR	Organophilic nano size clay	ethanol/water (5/95)	30	133.32	26.4	0.34	8.636	[95]
PVDF	Carbon black	2-propanol/water (4/96)	45	2399.80	6.34	4.18	22.3212	[52]
PIM	Functionalized GO	alcohol/water (5/95)	65	1000	32.9	0.930	29.667	[125]
PMPS	HOSSM-ZIF	Furfural(2-furancarboxaldehyde)/water (1/99)	100	-	42.9	1.40	58.7	[64]

**Table 9:** PV performance of organophilic nanocomposite membranes.

Polymer	Nanoparticle	Feed Solution and Composition (wt. Ratio)	Permeate	Operating Temperature (°C)	Downstream Pressure (Pa)	Separation Factor (α)	Total Flux (kg/m^2^.h)	PSI	Ref.
PVA	CS-wrapped MWNT	benzene/cyclohexane (50/50)	Benzene	50	1000	53.4	0.0659	3.4532	[34]
PDMS	SiO_2_	dimethyl carbonate (DMC)/methanol (30/70)	DMC	40	200	3.97	0.702	2.0849	[88]
	Clay	toluene/methanol (90/10)	Toluene	55	-	~2.1	~0.019	-	[32]
						~3.1	~0.0175	-	
PEBA	Cu^+^ and Fe^2+^ ions co-impregnated carbon nitride	thiophene-n-octane (thiophene content of 1312 ppm)	Thiophene	60	500	7.11	13.42	95.4162	[126]
PELSC		Methanol/MTBE (14.3/83.7)	MeOH	51	10799.11	89	1.23	108.24	[83]
poly (methyl methacrylate (MMA)-co-acrylamide (AM))	AgCl	benzene/cyclohexane (50/50)	Benzene	30	-	~ 9.25	~0.725	-	[132]
Poly (phenylene isophtalamide)	CNT	methanol/MTBE (15/85)	Methanol	50	13.33	~110	~0.667	-	[29]
CS	MWNTs-Ag^+^	benzene/cyclohexane (50/50)	Benzene	20	-	7.89	0.35796	2.4658	[30]
PPO	fullerene	acetic acid/ethanol/ethyl acetate/water (18.9/14.6/55.2/11.3)	Ethyl acetate	25	13.33	-	~ 0.8	~ 0.5	[50]
PEK-c	ZIF	water/methanol/MTBE (7.38/14.03/78.59)	water and methanol	50	25,000	19731	1.48 kg μm/m^2^h	25,000	[110]
